# Cellular pyrimidine imbalance triggers mitochondrial DNA–dependent innate immunity

**DOI:** 10.1038/s42255-021-00385-9

**Published:** 2021-04-26

**Authors:** Hans-Georg Sprenger, Thomas MacVicar, Amir Bahat, Kai Uwe Fiedler, Steffen Hermans, Denise Ehrentraut, Katharina Ried, Dusanka Milenkovic, Nina Bonekamp, Nils-Göran Larsson, Hendrik Nolte, Patrick Giavalisco, Thomas Langer

**Affiliations:** 1 Max Planck Institute for Biology of Ageing, Cologne, Germany; 2 Cologne Excellence Cluster on Cellular Stress Responses in Aging-Associated Diseases (CECAD), University of Cologne, Cologne, Germany; 3 Department of Medical Biochemistry and Biophysics, Karolinska Institutet, Stockholm, Sweden

**Keywords:** Innate immunity, Mitochondria, DNA metabolism

## Abstract

Cytosolic mitochondrial DNA (mtDNA) elicits a type I interferon response, but signals triggering the release of mtDNA from mitochondria remain enigmatic. Here, we show that mtDNA-dependent immune signalling via the cyclic GMP–AMP synthase‒stimulator of interferon genes‒TANK-binding kinase 1 (cGAS–STING–TBK1) pathway is under metabolic control and is induced by cellular pyrimidine deficiency. The mitochondrial protease YME1L preserves pyrimidine pools by supporting de novo nucleotide synthesis and by proteolysis of the pyrimidine nucleotide carrier SLC25A33. Deficiency of YME1L causes inflammation in mouse retinas and in cultured cells. It drives the release of mtDNA and a cGAS–STING–TBK1-dependent inflammatory response, which requires SLC25A33 and is suppressed upon replenishment of cellular pyrimidine pools. Overexpression of SLC25A33 is sufficient to induce immune signalling by mtDNA. Similarly, depletion of cytosolic nucleotides upon inhibition of de novo pyrimidine synthesis triggers mtDNA-dependent immune responses in wild-type cells. Our results thus identify mtDNA release and innate immune signalling as a metabolic response to cellular pyrimidine deficiencies.

## Abstract (web summary)

Sprenger et al. find that cytosolic pyrimidine levels regulate the release of mitochondrial DNA and activation of cGAS–STING.

## Main

Mitochondria serve as cellular signalling hubs and are intimately linked to innate immune signalling pathways^1,2^. Pathogen infection, apoptosis, chemotherapeutic agents or defective mtDNA packaging lead to the release of mtDNA into the cytosol^3–7^. Cytosolic mtDNA is recognized by specific receptor proteins, such as cyclic GMP–AMP synthase (cGAS), whose activation triggers the expression of specific interferon-stimulated genes (ISGs) via the adaptor protein stimulator of interferon genes (STING) and the downstream TANK-binding kinase 1 (TBK1)^8–10^. This immune response heightens antiviral immunity and protects against nuclear DNA damage^5^. Conversely, removal of dysfunctional mitochondria by mitophagy suppresses STING-dependent inflammation and neurodegeneration in mice, a mechanism that might be of relevance in Parkinson’s disease^11^.

The *i*-AAA protease YME1L, an ATP-dependent proteolytic complex in the mitochondrial inner membrane, coordinates mitochondrial biogenesis and dynamics with the metabolic output of mitochondria^12,13^. YME1L balances fusion and fission of mitochondria by processing of the dynamin-like GTPase optic atrophy 1 (OPA1) in response to metabolic cues^14–17^. Nutrient and oxygen availability regulate proteolytic rewiring of the mitochondrial proteome by YME1L tailoring mitochondria for efficient glutamine utilization if oxidative phosphorylation activity is limited^18^. YME1L-mediated metabolic reprogramming of mitochondria supports the growth of pancreatic ductal adenocarcinoma cells and may be relevant to the pathophysiology of these tumours^18^. Tissue-specific loss of YME1L in mice causes mitochondrial fragmentation and is associated with heart failure, disturbed eye development and axonal degeneration in the spinal cord^19,20^. In humans, mutations in *YME1L* cause a neurological disorder with ocular dysfunction and motor delay^21^. One of the earliest phenotypes of nervous-system-specific *Yme1l* knockout mice (NYKO mice) is an inflammatory response in the retina^19^, but why the loss of YME1L and impaired mitochondrial proteostasis induce inflammation remains unclear.

## Results

### Loss of YME1L elicits cGAS–STING–TBK1 immune signalling

**Fig. 1:**
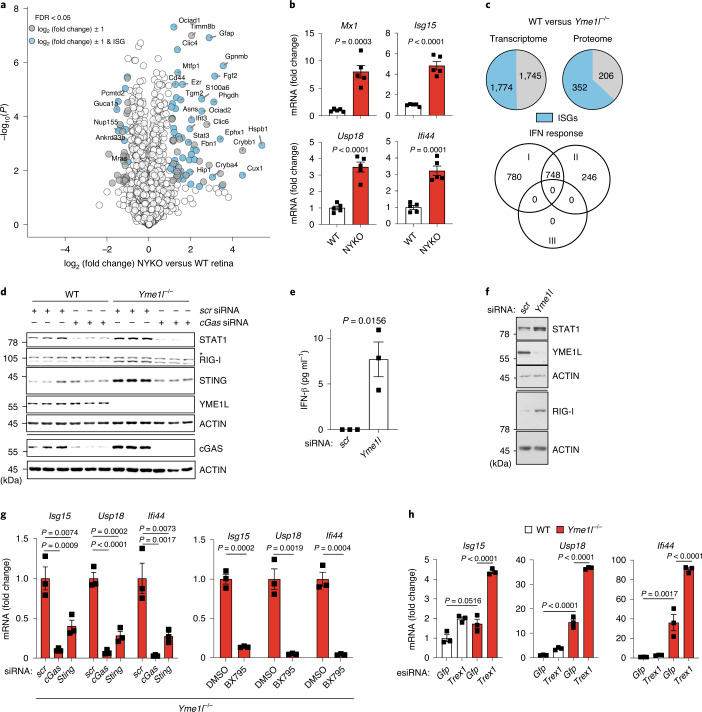
Loss of YME1L elicits an innate immune response along the cGAS–STING–TBK1 pathway. **a**, Quantitative proteomics of retinas from 31–32-week-old WT and NYKO mice (*n* = 5). ISGs with a NYKO versus WT log_2_ (fold change) of at least ±1 are shown in blue. Other proteins with a log_2_ ratio of at least ±1 are shown in grey. **b**, ISG expression in 6–7-week-old WT and NYKO retinas monitored by RT–qPCR (*n* = 5). **c**, Pie charts show ISGs (blue) (1774 in RNA-Seq; 352 in proteomics) among all significantly upregulated transcripts (3519) and proteins (558) in *Yme1l*^*−/−*^ versus WT MEFs (*n* = 3 independent cultures). Classification of the ISG transcripts into type I, II or III IFN responses according to the Interferome database. **d**, Immunoblot of MEFs treated with indicated siRNAs (*n* = 3 independent cultures). **e**, IFN-β enzyme-linked immunosorbent assay of cell culture supernatants from MEFs treated with the indicated siRNA (*n* = 3 independent cultures). **f**, Immunoblot of MEFs treated with the indicated siRNA (*n* = 3 independent cultures). **g**, ISG expression in MEFs treated with the indicated siRNAs or 0.1 µM of the TBK1 inhibitor BX795 for 72 h (*n* = 3 independent cultures). **h**, ISG expression in MEFs treated with the indicated esiRNAs for 72 h (*n* = 3 independent cultures). Knockdown controls are shown in Extended Data Fig. 1c,f. *P* values calculated using two-tailed unpaired *t*-test (**b**,**e**, right panel in **g**), one-way analysis of variance (ANOVA) with Dunnett’s multiple comparison test (left-hand panel in **g**) or two-way ANOVA with Tukey’s multiple comparison test (**h**). FDR, false discovery rate. Data are means ± s.e.m. Source data

**Extended Data Fig. 1:**
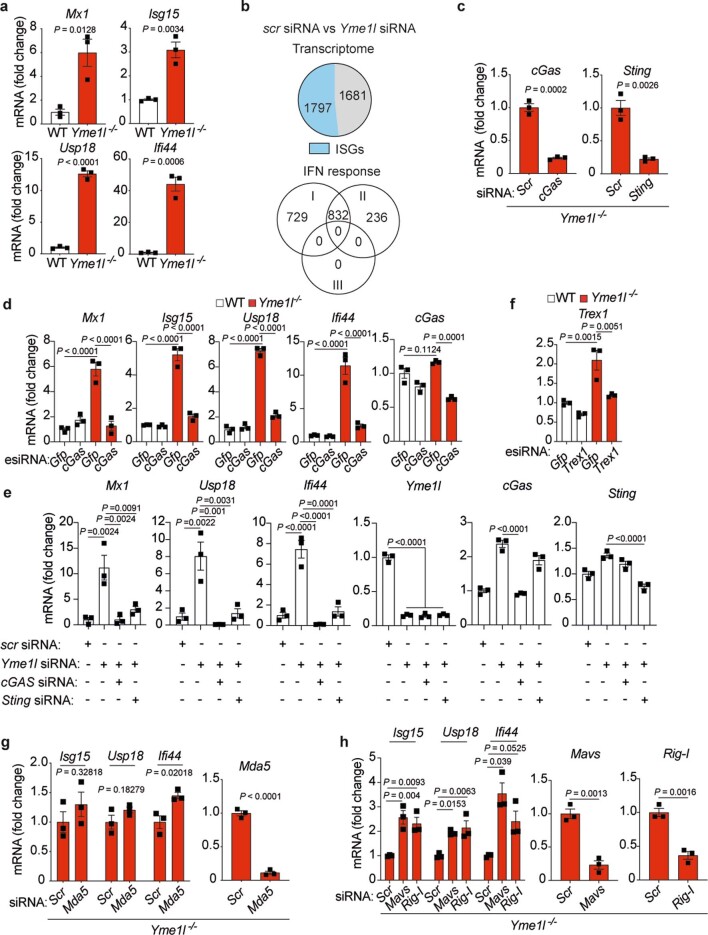
Innate immune signaling in *Yme1l*^-/-^ MEFs. **a**, ISG expression analyzed by qRT-PCR in wildtype (WT) and *Yme1l*^*-/-*^ MEFs (n=3 independent cultures). **b**, ISGs (blue, 1797) are highlighted among all significantly upregulated transcripts (3478) from RNA-Seq experiments in MEFs treated with scrambled (*Scr*) or siRNA against *Yme1l* (n=3 independent cultures). Venn diagram shows classification of all ISG transcripts into type I, II or III interferon responses (IFN) according to the Interferome database. **c-g**, qRT-PCR analysis to monitor ISG expression or knockdown efficiencies in MEFs treated with indicated siRNAs or esiRNAs. Immortalized MEFs were used in **c,d,f,g,h** (n=3 independent cultures). Primary MEFs were used in **e** (n=3 independent cultures). *P* values calculated using two-tailed unpaired t-test (**a,c,g**, right panels in **h**); two-way ANOVA with Tukey`s multiple comparison test (**d** and **f**); one-way ANOVA with Tukey`s multiple comparison test (**e**) Dunnett`s multiple comparison test (left panel in **h**). Data are means ± SEM.

To explore the inflammatory nature of YME1L-deficient tissues, we performed quantitative proteomics of retinas isolated from 31–32-week-old NYKO mice (Fig. 1a). The majority of proteins that accumulate in the retina of these mice are encoded by ISGs^22^, including the inflammatory marker protein glial fibrillary acidic protein (Fig. 1a). ISG expression was induced in the retinas of NYKO mice, as revealed by a quantitative polymerase chain reaction with reverse transcription (RT–qPCR) (Fig. 1b). Similarly, analysis of the transcriptome and proteome of mouse embryonic fibroblasts (MEFs) revealed that a large proportion of upregulated transcripts and proteins in *Yme1l*^*−/−*^ cells were encoded by ISGs and that the majority of these classified within the type I interferon (IFN) response (Fig. 1c and Extended Data Fig. 1a). These included ISGs involved in immune signalling, such as signal transducer and activator of transcription 1 (STAT1), cGAS, STING or retinoic acid inducible gene I (RIG-I), which all accumulated in *Yme1l*^*−*/*−*^ MEFs (Fig. 1d). Moreover, we observed significant upregulation of ISGs, increased level of STAT1 and secretion of IFNβ upon acute knockdown of *Yme1l* (Fig. 1e,f and Extended Data Fig. 1b).

Type I IFN responses are induced upon binding of cytosolic, double-stranded DNA (dsDNA) to cGAS, which activates ISG expression via the cGAS–STING–TBK1 pathway^8,9^. Depletion of cGAS or STING, as well as chemical inhibition of TBK1, dramatically reduced ISG expression in *Yme1l*^*−/−*^ cells (Fig. 1d,g and Extended Data Fig. 1c,d). Similarly, we observed cGAS- and STING-dependent ISG expression upon downregulation of *Yme1l* in primary MEFs (Extended Data Fig. 1e). By contrast, knockdown of the cytosolic three prime repair exonuclease 1 (TREX1) boosted ISG expression in YME1L-deficient cells (Fig. 1h and Extended Data Fig. 1f). This suggests that cytosolic dsDNA is responsible for ISG expression in *Yme1l*^*−/−*^ cells. Consistently, downregulation of the cytosolic RNA receptors melanoma differentiation-associated protein 5 (MDA5) and RIG-I or of the mitochondrial antiviral-signalling protein MAVS^23^ did not blunt ISG expression in *Yme1l*^*−/−*^ cells (Extended Data Fig. 1g,h). We therefore conclude that YME1L specifically protects against a dsDNA-dependent innate immune response along the cGAS–STING–TBK1 pathway.

### mtDNA release to the cytosol from mitochondria lacking YME1L

**Fig. 2:**
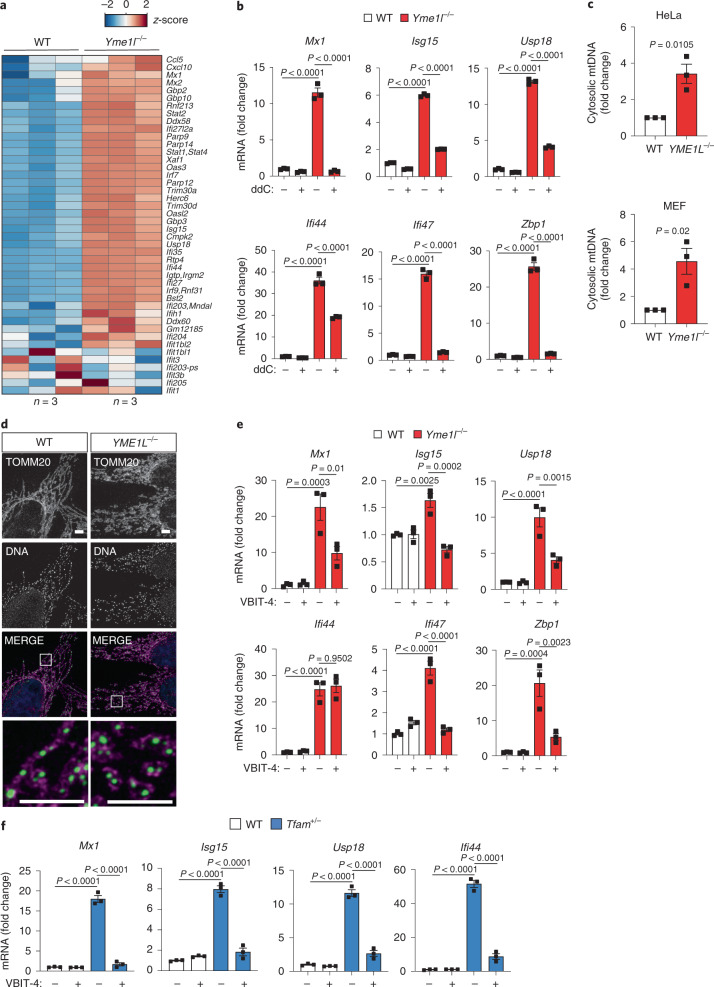
mtDNA is released into the cytosol in YME1L-deficient cells. **a**, Expression of ISGs, which were previously described as being induced by mtDNA stress upon depletion of TFAM (including in addition *Mx1*)^4,5^, in WT versus *Yme1l*^*−/−*^ MEFs (RNA-Seq; *z*-score normalized log_2_(FPKM) values, *n* = 3 independent cultures). **b**, ISG expression in MEFs treated with water or ddC (20 µM) for 9 days (*n* = 3 independent cultures). **c**, mtDNA levels in cytosolic fractions from HeLa cells and MEFs assessed by qPCR amplification of mitochondrial *CYTB* (*n* = 3 independent cultures). **d**, Immunocytochemistry of HeLa cells using antibodies against TOMM20 (mitochondria) and DNA, scale bar, 5 µm (*n* = 3 independent cultures). **e**, ISG expression in WT and *Yme1l*^*−/−*^ MEFs treated with 10 µM of the VDAC1 oligomerization inhibitor VBIT-4 for 48 h (*n* = 3 independent cultures). **f**, ISG expression in WT and *Tfam*^*−/−*^ MEFs treated with 10 µM VBIT-4 for 48 h (*n* = 3 independent cultures). *P* values calculated using two-way ANOVA with Tukey’s multiple comparison test (**b**,**e**,**f**) or two-tailed unpaired *t*-test (**c**). Data are means ± s.e.m.

**Extended Data Fig. 2:**
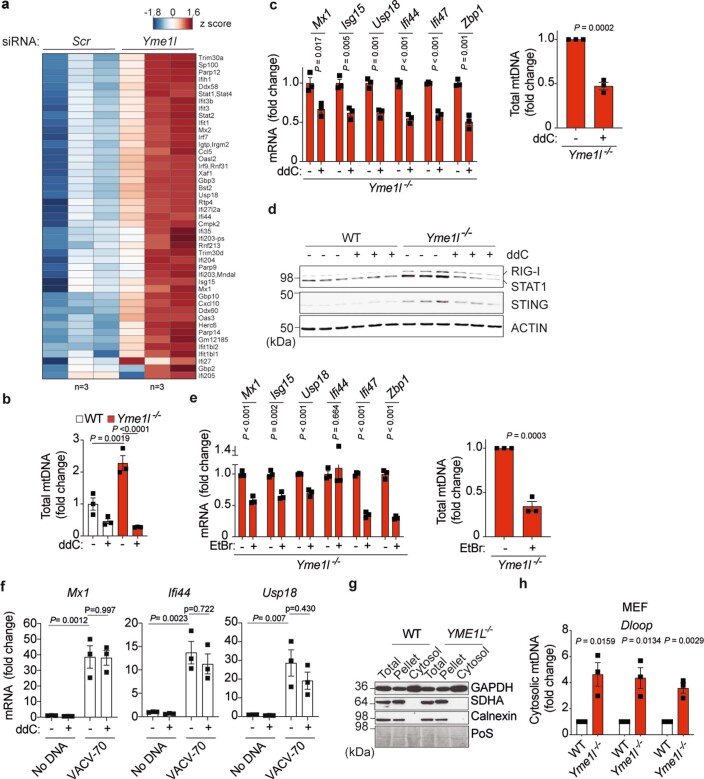
MtDNA release into the cytosol in YME1L-deficient cells. **a**, Heatmap of expression (RNA-Seq; z-score normalized log2 FPKM values) of ISGs, which were previously described to be induced by mtDNA stress upon depletion of TFAM (including in addition *Mx1*), in MEFs treated with scrambled (scr) or *Yme1l* siRNA (n=3 independent cultures). **b**, Total mtDNA levels analyzed by q-PCR amplification of *Cytb* in wildtype (WT) and *Yme1l*^-/-^ MEFs treated with 20 µM ddC for 9 days (n=3 independent cultures). **c**, ISG expression and total mtDNA levels monitored by q-PCR amplification of *Cytb* in *Yme1l*^-/-^ MEFs treated with 20 µM ddC for 72 h (n=3 independent cultures). **d**, Immunoblot analysis of cells treated as in (**c**) using antibodies directed against RIG-I, STAT1, STING, and, as loading control, β-ACTIN (n=3 independent cultures). **e**, ISG expression and total mtDNA (*Cytb)* in *Yme1l*^-/-^ MEFs treated with 300 ng/µl ethidium bromide (EtBr) for 72 h (n=3 independent cultures). **f**, ISG expression in WT MEFs transfected with 70bp oligonucleotides containing viral DNA motifs (VACV-70) after treatment with 40 µM ddC for 9 days (n=3 independent cultures). **g**, SDS-PAGE and immunoblot analysis of cytosolic fractions using antibodies directed against GAPDH (cytosol), SDHA (mitochondria) and calnexin (ER). Ponceau S (PoS) was used as a loading control (n=1). **h**, Expression levels of the *Dloop* region as a marker for mtDNA in cytosolic fractions from MEFs. Three different regions within the *Dloop* were amplified (n=3 independent cultures). *P* values calculated using two-way ANOVA with Tukey`s multiple comparison test (**b** and **f**); two-tailed unpaired t-test (**c,e,h**). Data are means ± SEM. Source data

Cytosolic dsDNA can originate from the nucleus and mitochondria. mtDNA-dependent innate immune signalling has been observed in *Tfam*^+/*−*^ cells harbouring reduced levels of mitochondrial transcription factor A (TFAM) that possibly could influence DNA packaging^4,5^. Notably, ISGs induced in YME1L-deficient MEFs largely overlap with those accumulating in TFAM-deficient MEFs suggesting the involvement of mtDNA (Fig. 2a and Extended Data Fig. 2a)^4,5^. To examine whether the innate immune response observed in YME1L-deficient cells depends on mtDNA, we depleted mtDNA from wild-type (WT) and *Yme1l*^*−*/*−*^ cells using 2′,3′-dideoxycytidine (ddC) or ethidium bromide (EtBr). Treatment with ddC efficiently depleted mtDNA from cells over time and resulted in strong suppression of ISG expression in *Yme1l*^*−*/*−*^ cells (Fig. 2b and Extended Data Fig. 2b–d), suggesting that cytosolic mtDNA may elicit the innate immune response in these cells. Consistently, ISG expression was also reduced after EtBr treatment of *Yme1l*^*−*/*−*^ cells, which lowered mtDNA levels (Extended Data Fig. 2e). For control, we transfected MEFs with viral dsDNA (VACV-70) and observed ISG expression irrespective of ddC treatment, demonstrating that the antiviral cGAS–STING–TBK1 response is intact in ddC-treated cells and can be mounted in the absence of mtDNA (Extended Data Fig. 2f). These experiments demonstrate mtDNA-dependent activation of cGAS in *Yme1l*^*−*/*−*^ cells. Indeed, cell fractionation experiments revealed that mtDNA accumulated in cytosolic fractions of MEFs and HeLa cells lacking YME1L (Fig. 2c and Extended Data Fig. 2g,h). Thus, YME1L has an anti-inflammatory role and prevents mtDNA release from mitochondria.

**Extended Data Fig. 3:**
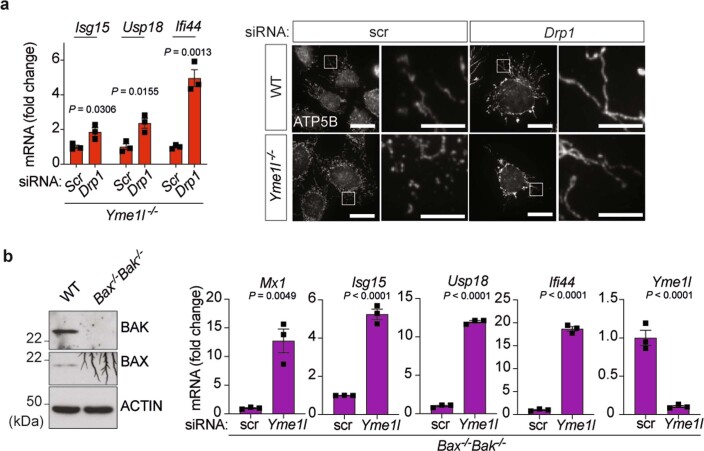
Innate immunity in YME1L-deficient cells is independent of mitochondrial fragmentation and BAX/BAK pores. **a**, ISG expression analyzed by qRT-PCR in *Yme1l*^-/-^ MEFs treated with the indicated siRNAs (left panel) (n=3 independent cultures). Analysis of MEFs by immunocytochemistry using antibodies directed against ATP5B (mitochondria) is shown in the right panel. Scale bar =20 µm; Scale bar in zoom images = 5 µm. (n=1) (**b**) Immunoblot analysis of WT and *Bax*^*-/-*^*Bak*^*-/-*^ MEFs (left panel) (n=1). qRT-PCR analysis to monitor ISG expression or knockdown efficiencies in *Bax*^*-/-*^*Bak*^*-/-*^ MEFs treated with indicated siRNAs (right) (n=3 independent cultures). *P* values calculated using two-tailed unpaired t-test. Data are means ± SEM. Source data

We next sought to understand how YME1L keeps mtDNA within mitochondria. Loss of YME1L leads to fragmentation of the mitochondrial network and increases the vulnerability towards cell death^15,19,20^. Whereas tubular mitochondria were restored upon depletion of the mitochondrial fission factor DRP1, ISG expression increased further in *Yme1l*^*−/−*^ cells depleted of DRP1 (Extended Data Fig. 3a). The stimulation of ISGs in the absence of YME1L is therefore independent of mitochondrial fragmentation. This is in line with previous results demonstrating that fusion mediated by mitofusin 1 is required for ISG expression in *Tfam*^+/*−*^ cells^4^ and that *Opa1*^*−/−*^ muscle or T cells do not show an exaggerated ISG response^24,25^. Notably, restoration of tubular mitochondria suppresses the apoptotic sensitivity of *Yme1l*^*−*/*−*^ cells^15^, suggesting that distinct mechanisms trigger ISG expression and apoptosis in *Yme1l*^*−*/*−*^ cells. In apoptotic cells, mtDNA is ejected from mitochondria via herniation of the mitochondrial inner membrane through Bcl-2-associated X protein/Bcl-2 homologous antagonist/killer (BAX/BAK) pores in the mitochondrial outer membrane^6,7,26–28^. However, depletion of YME1L boosted ISG expression in *Bax*^*−/−*^*Bak*^*−/−*^ MEFs (Extended Data Fig. 3b). In contrast to apoptotic cells, immunocytochemistry did not show any change in nucleoid structure or accumulation of DNA nucleoids in the cytosol of *Yme1l*^*−/−*^ cells (Fig. 2d). Because mtDNA fragments can be released from mitochondria upon oligomerization of voltage-dependent anion channels (VDAC) 1/3 (ref. ^3^), we treated *Yme1l*^*−*/*−*^ cells with the VDAC inhibitor VBIT-4 and monitored ISG expression. VBIT-4 impaired expression of most tested ISGs in *Yme1l*^*−*/*−*^ cells as well as in *Tfam*^+/*−*^ cells, suggesting that mtDNA is released from mitochondria lacking YME1L through VDAC pores (Fig. 2e,f).

### SLC25A33 drives mtDNA-dependent innate immunity

**Fig. 3:**
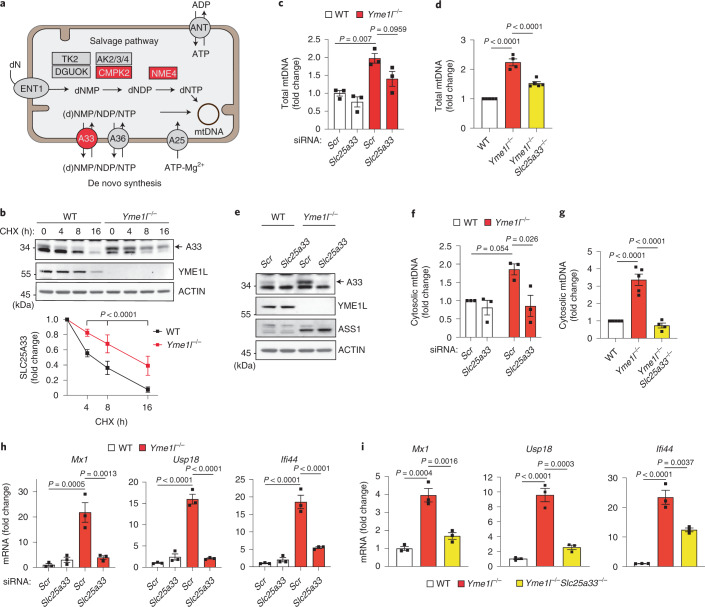
Proteolysis of SLC25A33 by YME1L controls mtDNA-dependent innate immunity. **a**, Nucleotide synthesis via mitochondrial salvage or cytosolic de novo pathway. Components that affect the metabolism of mtDNA and that accumulate in *Yme1l*^*−*/*−*^ cells are highlighted in red. **b**, Cycloheximide (CHX) treatment of MEFs for the indicated time. Quantification of SLC25A33 levels is shown in the lower panel (mean ± SD; *n* = 7 independent cultures). **c**,**d**, Total mtDNA level monitored by qPCR (*Cytb*) in WT and *Yme1l*^*−*/*−*^ MEFs treated with the indicated siRNAs (*Slc25a33* no. 1) (*n* = 3 independent cultures) (**c**) or in WT, *Yme1l*^*−/−*^ and *Yme1l*^*−/−*^*Slc25a33*^*−/−*^ MEFs (*n* = 5 independent cultures for WT and *Yme1l*^*−/−*^*Slc25a33*^*−/−*^; *n* = 4 independent cultures for *Yme1l*^*−/−*^ (**d**). **e**, Immunoblot analysis of WT and *Yme1l*^*−/−*^ MEFs treated with the indicated siRNAs (*Slc25a33* no. 1) (representative blot from *n* = 3 independent cultures). **f**,**g**, mtDNA levels in cytosolic fractions monitored by qPCR (*Cytb*) in WT and *Yme1l*^*−*/*−*^ MEFs treated with the indicated siRNAs (*Slc25a33* no. 1) (*n* = 3 independent cultures) (**f**) or in WT, *Yme1l*^*−/−*^ and *Yme1l*^*−/−*^*Slc25a33*^*−/−*^ MEFs (*n* = 5 independent cultures for WT and *Yme1l*^*−/−*^; *n* = 4 independent cultures for *Yme1l*^*−/−*^*Slc25a33*^*−/−*^) (**g**). **h**,**i**, ISG expression in WT and *Yme1l*^*−*/*−*^ MEFs treated with the indicated siRNAs (*Slc25a33* no. 2) (*n* = 3 independent cultures) (**h**) or in WT, *Yme1l*^*−/−*^ and *Yme1l*^*−/−*^*Slc25a33*^*−/−*^ MEFs (*n* = 3 independent cultures) (**i**). *P* values calculated using two-tailed multiple *t*-test with Holm–Sidak method to correct for multiple comparisons (**b**), two-way ANOVA with Tukey’s multiple comparison test (**c**,**f**,**h**) or one-way ANOVA with Tukey’s multiple comparison test (**d**,**g**,**i**). Data (except **b**) are means ± s.e.m. Source data

**Extended Data Fig. 4:**
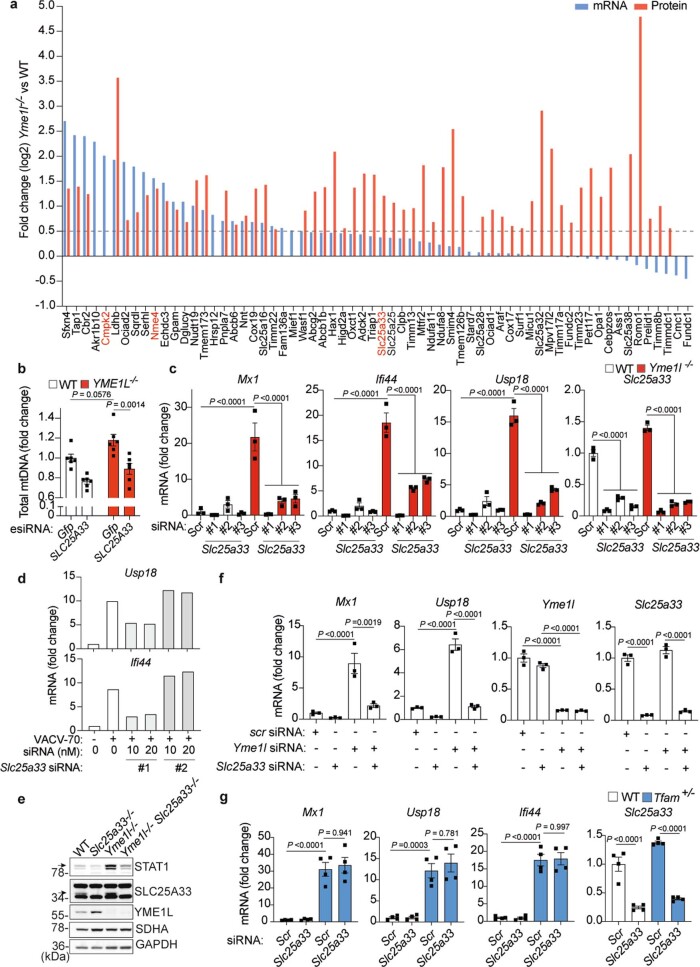
Gene expression analysis and SLC25A33-dependent ISG expression in YME1L-deficient cells. **a**, RNA-Seq analysis of proteins accumulating in *Yme1l*^-/-^ MEFs (n=3 independent cultures) identified by quantitative mitochondrial proteomics (*14*). Log2 transformed fold changes of mRNA and protein levels are shown. Cmpk2, Nme4 and Slc25a33 are highlighted in red and illustrated in Fig. 3a. The fold change of some proteins (for example Cmpk2 and Ass1) was not determined since they were detected exclusively in *Yme1l*^-/-^ mitochondria (*14*). **b**, Total mtDNA levels (*CYTB)* monitored by q-PCR in wildtype (WT) and *YME1L*^-/-^ HeLa treated with indicated esiRNAs (n=6 independent cultures). **c**, ISG and *Slc25a33* expression in WT and *Yme1l*^-/-^ MEFs treated with the indicated siRNAs (Data for *Scr* and *Slc25a33#2* siRNAs are the same as shown in Fig. 3h) (n=3 independent cultures). **d**, ISG expression in cells treated with 2µg of VACV-70 viral DNA motif for 4 h after 72 h incubation with the indicated siRNAs (n=1). **e**, Immunoblot analysis of WT, *Slc25a33*^*-/-*^, *Yme1l*^*-/-*^ and *Yme1l*^*-/-*^*Slc25a33*^*-/-*^MEFs (n=1). Arrows indicate STAT1 and SLC25A33 protein level, respectively. **f**, ISG expression monitored by qRT-PCR in primary WT MEFs treated with indicated siRNAs (*Slc25a33* #2) (n=3 independent cultures). **g**, ISG expression in WT and *Tfam*^+/-^ MEFs treated with indicated siRNAs (*Slc25a33* #2) (n=4 independent cultures). *P* values calculated using two-way ANOVA with Tukey`s multiple comparison test (**b, c** and **g**) and one-way ANOVA with Tukey`s multiple comparison test (**f**). Data are means ± SEM. Source data

Our recent proteomic survey of *Yme1l*^*−*/*−*^ cells revealed broad rewiring of the mitochondrial proteome and the accumulation of a number of mitochondrial proteins, including YME1L substrate proteins^18^. Notably, several of these proteins are associated with mtDNA metabolism (Fig. 3a and Extended Data Fig. 4a), namely the pyrimidine nucleotide carrier SLC25A33 and components of the mitochondrial nucleoside salvage pathway, the cytidine/uridine monophosphate kinase 2 (CMPK2) and the nucleoside diphosphate kinase NME4 (Fig. 3a and Extended Data Fig. 4a). Whereas CMPK2 and NME4 are transcriptionally upregulated in *Yme1l*^*−/−*^ cells (Extended Data Fig. 4a), cycloheximide chase experiments revealed an increased stability of SLC25A33 in *Yme1l*^*−/−*^ cells, suggesting that it is a YME1L substrate protein (Fig. 3b). The moderate stabilization of SLC25A33 in *Yme1l*^*−*/*−*^ cells indicates that an additional protease can mediate proteolysis of this metabolite carrier in the absence of YME1L (Fig. 3b). Because nucleotide transport across the mitochondrial inner membrane is linked to mtDNA maintenance^29,30^, we monitored the accumulation of mtDNA in WT and *Yme1l*^*−/−*^ cells upon depletion of *Slc25a33*. We observed moderately elevated mtDNA levels in YME1L-deficient MEFs and HeLa cells (Fig. 3c and Extended Data Fig. 4b). Depletion of *Slc25a33* decreased mtDNA levels in *Yme1l*^*−*/*−*^ cells (Fig. 3c and Extended Data Fig. 4b). These results were corroborated by deletion of *Slc25a33* in *Yme1l*^*−*/*−*^ cells (Fig. 3d). Thus, the pyrimidine carrier SLC25A33 is required for the accumulation of mtDNA in cells lacking YME1L. These findings are consistent with the notion that nucleotide uptake into mitochondria limits mtDNA synthesis^29,30^.

**Extended Data Fig. 5:**
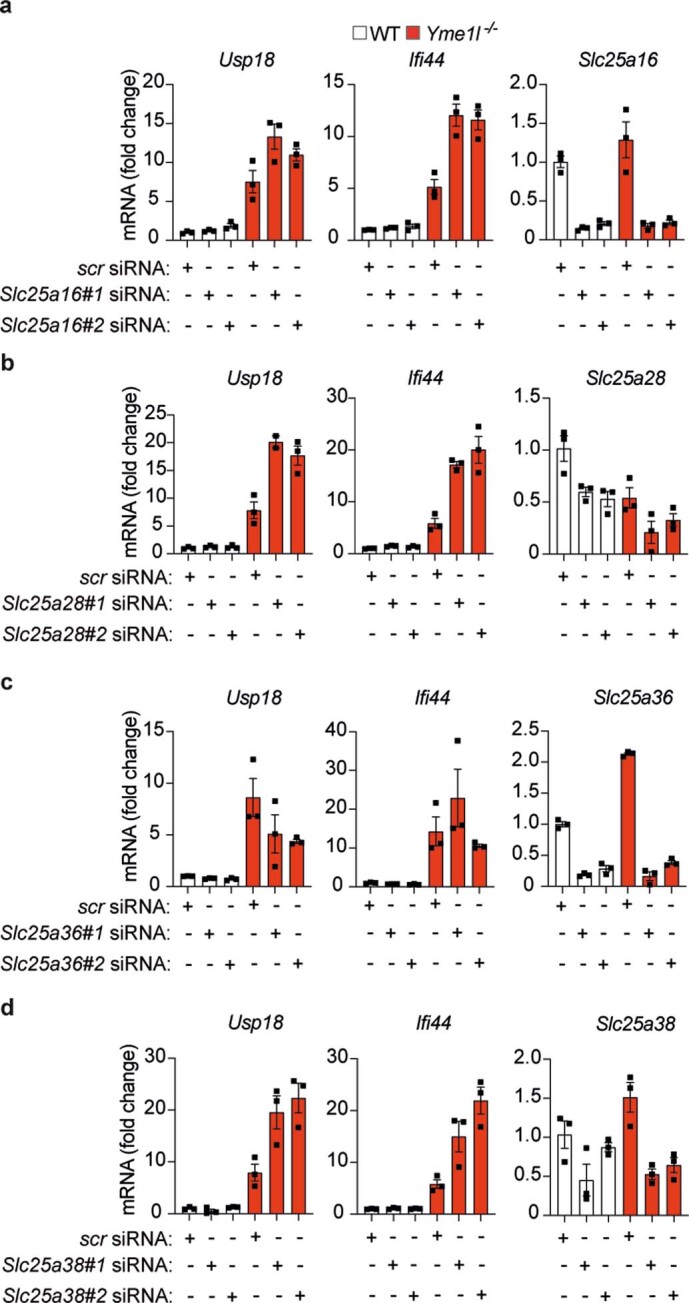
ISG expression in WT and YME1L-deficient MEFs treated with siRNAs against different SLC25 carrier proteins. **a-d**, ISG expression monitored by qRT-PCR in WT and *Yme1l*^*-/-*^ MEFs treated with indicted siRNAs (n=3 independent cultures). Data are means ± SEM.

To examine whether SLC25A33 affects mtDNA release from YME1L-deficient mitochondria, we analysed the accumulation of mtDNA in the cytosol of *Yme1l*^*−*/*−*^ cells depleted of SLC25A33. The loss of SLC25A33 reduced cytosolic mtDNA levels in *Yme1l*^*−*/*−*^ cells (Fig. 3f,g). Consistently, both knockdown or deletion of *Slc25a33* suppressed ISG expression in *Yme1l*^*−/−*^ cells (Fig. 3h,i and Extended Data Fig. 4c–e). Similar observations were made in primary MEFs (Extended Data Fig. 4f). By contrast, downregulation of other solute carriers accumulating in *Yme1l*^*−*/*−*^ cells did not inhibit ISG expression in *Yme1l*^*−*/*−*^ cells (Extended Data Fig. 5a–d). We therefore conclude that the mtDNA-dependent innate immune response in YME1L-deficient cells depends on SLC25A33 and pyrimidine transport between the cytosol and mitochondria. Although SLC25A33 likely affects ISG activation by mediating pyrimidine transport between the cytosol and mitochondria, a direct role of SLC25A33 in mtDNA release cannot be excluded. However, depletion of SLC25A33 did not impair ISG expression in *Tfam*^+/*−*^ cells indicating that SLC25A33 is not required for mtDNA release in these cells which appears to be triggered by different mechanisms (Extended Data Fig. 4g).

**Fig. 4:**
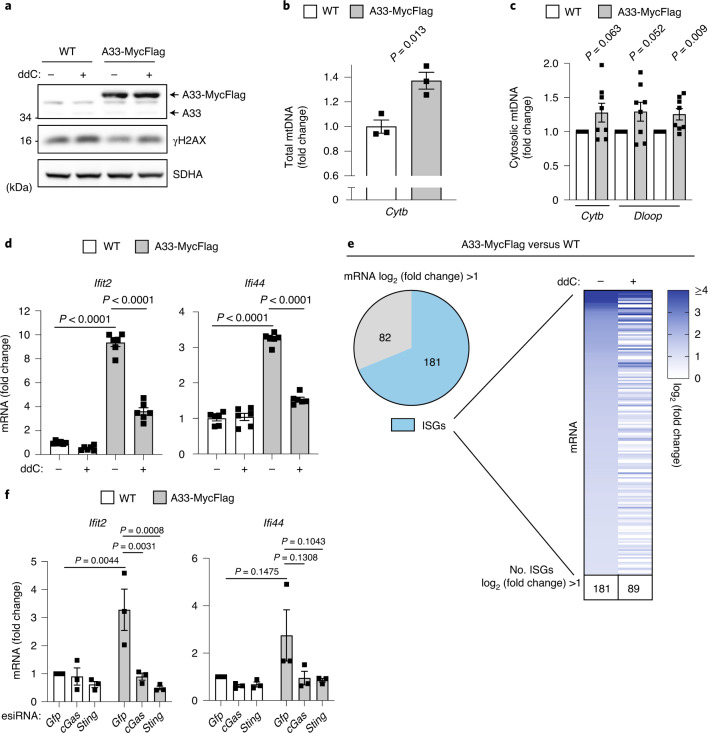
SLC25A33 overexpression is sufficient to induce mtDNA-dependent innate immunity. **a**, Immunoblot of WT and SLC25A33-Myc-Flag (A33-MycFlag) expressing HeLa cells treated with 40 µM ddC for 72 h (*n* = 1). **b**,**c**, Total and cytosolic mtDNA level in WT and SLC25A33–MycFlag (A33-MycFlag) expressing HeLa cells (*n* = 3 independent cultures (**b**) and *n* = 8 independent cultures (**c**)). **d**,**e**, ISG expression in WT and A33-MycFlag expressing HeLa cells treated with 40 µM ddC for 72 h analysed by RT–qPCR (**d**, *n* = 6 independent cultures) and RNA-Seq (**e**, *n* = 3 independent cultures). One hundred and eighty-one ISGs of 263 genes are upregulated (log_2_(fold change) ≥1) in A33-MycFlag versus WT cells in the absence of ddC. The heatmap depicts the relative expression of 181 ISGs in ddC-treated A33-MycFlag versus ddC-treated WT cells. **f**, ISG expression in WT and A33-MycFlag expressing HeLa cells treated with indicated esiRNAs (*n* = 3 independent cultures). *P* values calculated using two-tailed unpaired *t*-test (**b**,**c**) or two-way ANOVA with Tukey’s multiple comparison test (**d**,**f**). Data are means ± s.e.m. Source data

To examine whether the accumulation of SLC25A33 is sufficient to drive a mtDNA-dependent innate immune response, we overexpressed SLC25A33 in HeLa cells (Fig. 4a). We observed a moderate increase in total and cytosolic mtDNA (Fig. 4b,c) and increased ISG expression (Fig. 4d,e), which was dependent on cGAS and STING (Fig. 4f). Moreover, the inflammatory response was ameliorated upon ddC treatment of the cells illustrating the contribution of mtDNA (Fig. 4d,e). Genome-wide expression analysis of SLC25A33 overexpressing cells showed that ~70% of significantly upregulated genes are ISGs and that ~50% of them are induced in an mtDNA-dependent manner (Fig. 4e). We conclude that increased mitochondrial nucleotide transport by SLC25A33 is necessary and sufficient to elicit an innate immune response triggered by mtDNA. YME1L proteolysis limits the accumulation of SLC25A33 and thereby suppresses this response.

### YME1L preserves cellular nucleotide pools

**Fig. 5:**
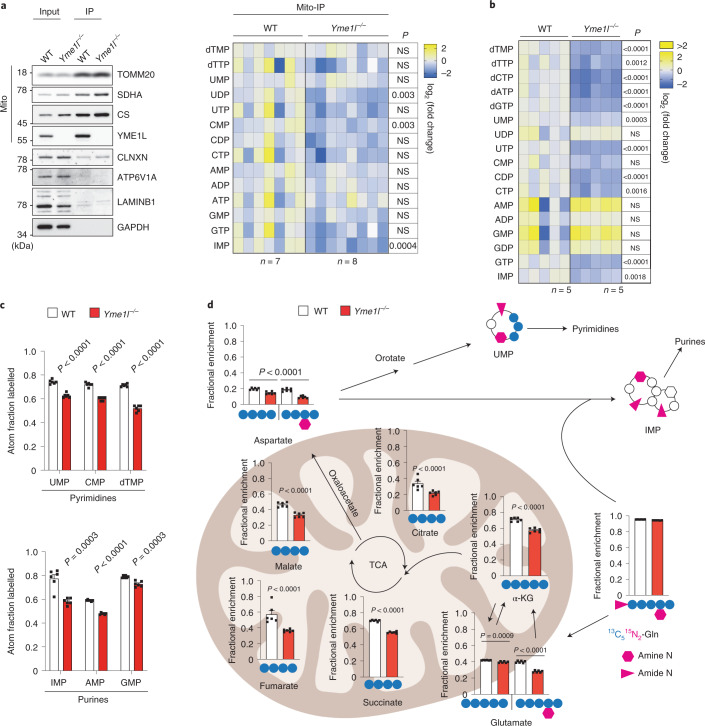
YME1L supports de novo nucleotide synthesis and maintains cellular nucleotide balance. **a**, Immunoblot analysis of isolated mitochondria using HA-MITO-expressing MEFs (left) and a heatmap showing log_2_(fold change) in the indicated nucleotide levels in *Yme1l*^*−/−*^ MEFs (*n* = 8 independent cultures) compared with WT (*n* = 7 independent cultures). Each square represents an individual replicate. **b**, Heatmap of log_2_(fold change) in the indicated nucleotide levels in *Yme1l*^*−/−*^ compared with WT. Each square represents an individual replicate (*n* = 5 independent cultures). **c**, The atom fraction enrichment of glutamine-derived ^13^C and/or ^15^N in nucleotides after treatment of WT and *Yme1l*^*−*/*−*^ MEFs with 2 mM ^13^C_5_^15^N_2_-glutamine for 6 h (*n* = 6 independent cultures). Mass isotopologue distribution within each nucleotide species are shown in Extended Data Fig. 6a,b and Supplementary Table 1. **d**, Graphical representation of ^13^C_5_^15^N_2_-glutamine flux in and out of the TCA cycle via glutaminolysis in WT and *Yme1l*^*−/−*^ MEFs cultured for 30 min in medium containing 2 mM ^13^C_5_^15^N_2_-glutamine (*n* = 6 independent cultures). The main isotopologue(s) of each metabolite are indicated below each chart and plotted as the fraction of the sum of all isotopologues. All enriched isotopologues are shown in Extended Data Fig. 6c and Supplementary Table 1. *P* values calculated using two-tailed multiple *t*-test with Holm–Sidak method to correct for multiple comparisons (**a**,**b**,**c**) or two-tailed unpaired *t*-test (**d**). NS, not significant; TCA, tricarboxylic acid cycle. Data are means ± s.e.m. Source data

**Extended Data Fig. 6:**
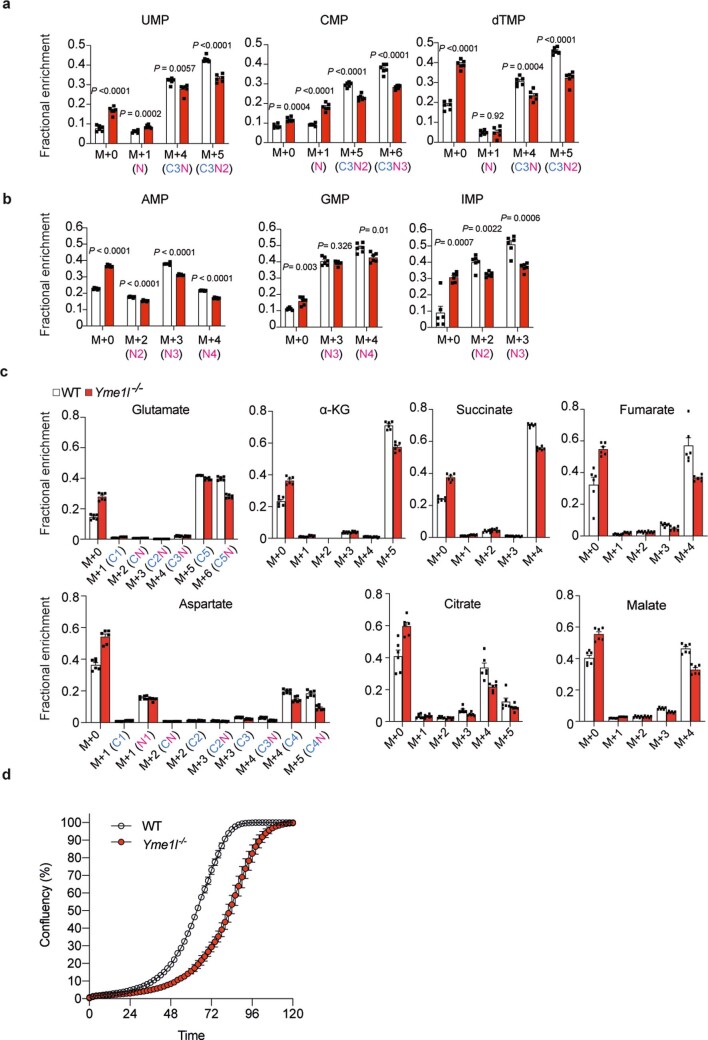
Cell growth and mass isotopologue anaylsis in WT and *Yme1l*^-/-^ cells. **a,b**, Mass isotopologue distribution in pyrimidines (**a**) and purines (**b**) from WT and *Yme1l*^*-/-*^ MEFs cultured for 6 h in medium containing 2 mM ^13^C_5_,^15^N_2_ glutamine (n=6 independent cultures). Each isotopologue is shown as a fraction of the sum of all possible isotopologues. **c**, Mass isotopologue distribution in the indicated metabolites after 30 min labelling with 13C_5_, 15N_2_-Glutamine in WT and *Yme1l*^*-/-*^ MEFs. For glutamate and aspartate, the presence and number of assimilated ^13^C and/or ^15^N in each metabolite isotopologue fraction is shown. For the other metabolites, isotopologue masses (M) are determined by the number of assimilated ^13^C. Selected isotopologues of each metabolite with statistical analysis are shown in Fig. 5d (n=6 independent cultures) and all metabolite isotopolgues are presented in Supplementary Table 1. **d**, Cell growth of WT and *Yme1l*^*-/-*^ MEFs monitored in IncuCyte S3 live-cell analysis system (n=3 independent cultures). *P* values calculated using two-tailed multiple t-test with Holm-Sidak method to correct for multiple comparisons (**a,b**). Data are means ± SEM.

To further characterize the role of YME1L in nucleotide and mtDNA metabolism, we determined cellular and mitochondrial nucleotide levels in WT and *Yme1l*^*−/−*^ MEFs. Quantitative metabolomics revealed a broad depletion of nucleotides with pyrimidines being predominantly affected (Fig. 5a,b). This holds true for both *Yme1l*^*−/−*^ cells and their mitochondria, which were rapidly purified from MEFs expressing the recently described HA-MITO-Tag in the mitochondrial outer membrane^31^ (Fig. 5a,b). Thus, despite the increased levels of SLC25A33 and the transcriptional activation of components of the mitochondrial nucleoside salvage pathway, nucleotides did not accumulate within mitochondria. It is conceivable that imported nucleotides are efficiently incorporated into mtDNA, in agreement with the moderately increased mtDNA levels in YME1L-deficient and SLC25A33 overexpressing cells (Figs. 3c,d and 4b). Alternatively, and not mutually exclusively, loss of YME1L may decrease the cytosolic de novo synthesis of nucleotides. Consistently, YME1L-mediated proteolysis ensures efficient utilization of glutamine^18^, which is a source of carbon and nitrogen for de novo pyrimidine synthesis and a source of nitrogen for de novo purine synthesis^32^. We traced stable isotope-labelled ^13^C_5_^15^N_2_-glutamine in WT and *Yme1l*^*−*/*−*^ MEFs and observed reduced labelling of pyrimidine and purine nucleotides in the absence of YME1L (Fig. 5c and Extended Data Fig. 6a,b). The incorporation of labelled carbon from glutamine into citric acid cycle metabolites via glutaminolysis was also perturbed (Fig. 5d and Extended Data Fig. 6c), substantiating the requirement of YME1L for efficient glutamine utilization^18^. Together, these findings reveal a dual role of YME1L in nucleotide homeostasis: it is required for efficient glutaminolysis and de novo nucleotide synthesis in the cytosol and regulates the uptake of pyrimidine nucleotides into mitochondria.

**Extended Data Fig. 7:**
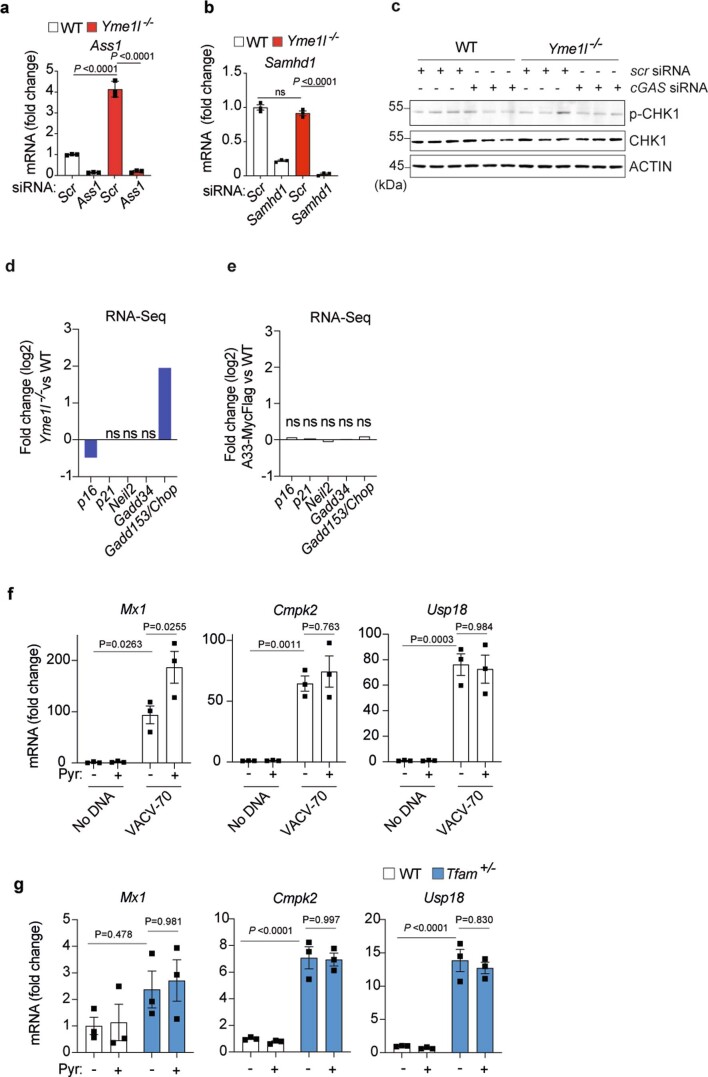
No evidence of DNA damage in YME1L-deficient cells. **a-b**, Expression levels of *Ass1* and *Samhd1* monitored by qRT-PCR in WT and *Yme1l*^*-/-*^ MEFs treated with the indicated siRNAs (n=3 independent cultures). **c**, Immunoblot analysis of WT and *Yme1l*^*-/-*^ MEFs treated with indicated siRNAs (n=3 independent cultures). **d-e**, Log2 transformed fold changes of mRNA (RNA-Seq) of previously published DNA damage response (DDR) associated genes in *Yme1l*^-/-^ compared to WT MEFs (n=3 independent cultures) (**d**) and in HeLa cells expressing SLC25A33-MycFlag compared to WT (n=3 independent cultures) (**e**). **f**, ISG expression in WT MEFs cultured in the presence or absence of pyrimidine nucleosides (Pyr) and transfected with 2 µg of VACV-70 for 4 h (n=3 independent cultures). **g**, ISG expression in WT and *Tfam*^+/-^ MEFs cultured in the presence or absence of pyrimidine nucleosides (n=3 independent cultures). *P* values calculated using two-way ANOVA with Tukey`s multiple comparison test (**a,b,f,g**). **d-e**, Statistical significance was determined using Benjamini–Hochberg method. p-values p<0.05 were considered to be significant. ns= not significant. Data are means ± SEM. Source data

In line with its critical role in nucleotide metabolism, loss of YME1L reduces cell growth as revealed by cell proliferation experiments (Extended Data Fig. 6d). Although nucleotide depletion induces nuclear DNA damage and can lead to a cGAS-dependent innate immune response^8^, the DNA damage response pathway is not activated in *Yme1l*^*−*/*−*^ MEFs, as monitored by RNA-Seq and phosphorylation of checkpoint kinase 1, which is a marker for DNA damage^33^ (Extended Data Fig. 7c,d). Similarly, no evidence of a DNA damage response was detected in cells overexpressing SLC25A33 (Fig. 4a and Extended Data Fig. 7e). This observation agrees with previous findings in *Tfam*^+/*−*^ cells, demonstrating that increased ISG expression upon mtDNA stress enhances nuclear DNA repair and protects the genome^5^.

### Pyrimidine deficiency causes mtDNA-dependent ISG expression

**Fig. 6:**
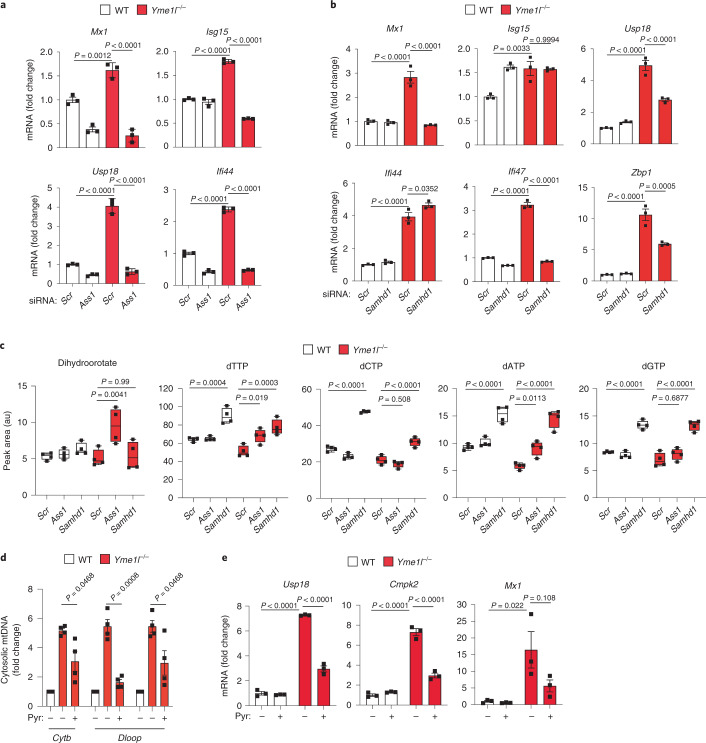
Rebalancing pyrimidine metabolism in YME1L-deficient cells protects against mtDNA release and inflammation. **a**, ISG expression determined by RT–qPCR in WT and *Yme1l*^*−/−*^ MEFs treated with the indicated siRNAs (*n* = 3 independent cultures). **b**, ISG expression determined by RT–qPCR in WT and *Yme1l*^*−/−*^ MEFs treated with the indicated siRNAs (*n* = 3 independent cultures). **c**, Dihydroorotate and nucleotide levels determined by mass spectrometry (**c**) in WT and *Yme1l*^*−*/*−*^ MEFs treated with the indicated siRNAs. Centre lines denote medians; box limits denote 25th and 75th percentiles; whiskers denote maxima and minima (*n* = 4 independent cultures). **d**,**e**, mtDNA levels in cytosolic fractions monitored by qPCR (*n* = 4 independent cultures) (**d**) and ISG expression by RT–qPCR (*n* = 3 independent cultures) (**e**) in WT and *Yme1l*^*−*/*−*^ MEFs cultured in the presence or absence of pyrimidine nucleosides (Pyr; 100 µM cytidine, thymidine and uridine). *P* values calculated using two-way ANOVA with Tukey’s multiple comparison test (**a**–**c**,**e**) or two-tailed unpaired *t*-test (**d**). Data are means ± s.e.m.

To explore a possible link between impaired nucleotide synthesis and the innate immune response in *Yme1l*^*−/−*^ cells, we first tested whether replenishing the cytosolic nucleotide pool in YME1L-deficient cells by genetic intervention can blunt ISG expression. Argininosuccinate synthetase 1 is transcriptionally upregulated in *Yme1l*^*−/−*^ cells (Fig. 3e and Extended Data Fig. 4a)^18^, where it contributes to the depletion of pyrimidine nucleotides by diverting aspartate from pyrimidine synthesis towards the urea cycle^34^. Accordingly, depletion of argininosuccinate synthetase 1 increased nucleotide levels and completely abolished ISG expression in *Yme1l*^*−/−*^ cells (Fig. 6a,c and Extended Data Fig. 7a). Similarly, increasing cellular deoxynucleoside triphosphate pools upon depletion of the deoxynucleoside triphosphate triphosphohydrolase, SAMHD1 (ref. ^35^), suppressed the innate immune response in *Yme1l*^*−*/*−*^ cells (Fig. 6b,c and Extended Data Fig. 7b).

Because YME1L regulates pyrimidine transport via SLC25A33 and chiefly cytosolic pyrimidine levels were diminished upon depletion of YME1L (Fig. 4b), we next tested whether exogenous supply of pyrimidines could blunt the innate immune response in YME1L-deficient cells. Strikingly, supplementation of these cells with a combination of thymidine, cytidine and uridine was sufficient to significantly reduce cytosolic mtDNA levels in *Yme1l*^*−*/*−*^ cells and to suppress ISG expression (Fig. 6d,e). Pyrimidine supplementation meanwhile had neither an effect on cGAS activation by VACV-70 (Extended Data Fig. 7f) nor on ISG expression in *Tfam*^+/*−*^ cells (Extended Data Fig. 7g). Thus, both the described genetic interventions and the pyrimidine complementation studies highlight the importance of pyrimidine deficiencies for the induction of mtDNA release and the immune response in cells lacking YME1L.

### Impaired pyrimidine synthesis drives mtDNA immune signalling

**Fig. 7:**
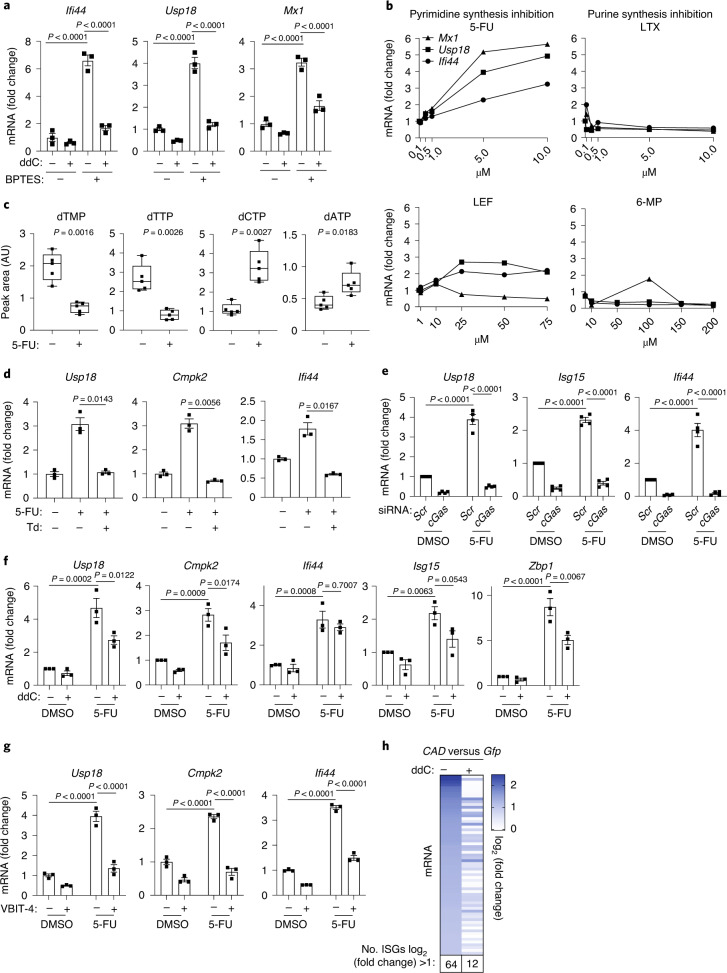
Inhibition of cytosolic pyrimidine metabolism induces mtDNA-dependent innate immunity. **a**, ISG expression in WT MEFs treated with water or 20 µM ddC for 9 days followed by 30 µM BPTES for 24 h (*n* = 3 independent cultures). **b**, ISG expression in WT MEFs treated with pyrimidine or purine synthesis inhibitors for 16 h (*n* = 1). LTX, lometrexol hydrate; LEF, leflunomide; 6-MP, 6-mercaptopurine. **c**, Nucleotide level in WT MEFs treated with 5 µM 5-FU for 16 h. Centre lines denote medians; box limits denote 25th and 75th percentiles; whiskers denote maxima and minima (*n* = 5 independent cultures). **d**, ISG expression in WT MEFs treated with a combination of 5 µM 5-FU and 200 µM thymidine (Td) for 16 h (*n* = 3 independent cultures). **e**,**f**, ISG expression in WT MEFs treated with indicated siRNAs (*n* = 4 independent cultures) (**e**) or 40 µM ddC for 9 days followed by 5 µM 5-FU for 16 h (*n* = 3 independent cultures) (**f**). **g**, ISG expression in WT MEFs treated with 10 µM of the VDAC1 oligomerization inhibitor VBIT-4 for 48 h followed by 5 µM 5-FU for 16 h (*n* = 3 independent cultures). **h**, RNA-Seq analysis of HeLa cells treated with *Gfp* or *CAD* esiRNA in the absence of ddC identified 64 ISGs upregulated upon CAD depletion (log_2_(fold change) ≥1). The heatmap depicts the relative expression of 64 ISGs in CAD-depleted cells that were treated or not treated with ddC (*n* = 3 independent cultures). *P* values calculated using two-way ANOVA with Tukey’s multiple comparison test (**a**,**e**–**g**) or two-tailed unpaired *t*-test (**c**,**d**). AU, arbitrary units. Data are means ± s.e.m.

**Extended Data Fig. 8:**
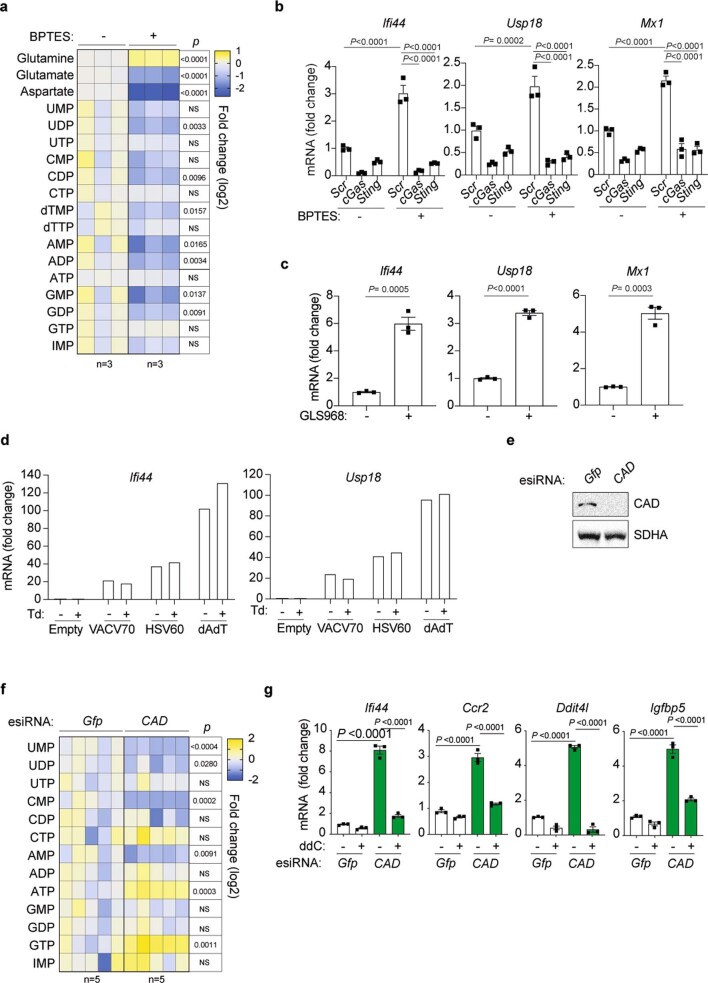
Cellular nucleotide metabolism is linked to ISG expression. **a**, Nucleotide level in WT MEFs treated with 30 µM BPTES for 4 h (n=3 independent cultures). **b**, ISG expression in WT MEFs treated with indicated siRNAs and 30 µM BPTES for 24 h (n=3 independent cultures). **c**, ISG expression in WT MEFs treated with 25 µM GLS968 for 24 h (n=3 independent cultures). **d**, ISG expression in WT MEFs treated with 200 µM thymidine (Td) for 48 h and transfected with 2 µg of the indicated dsDNA motifs for 4 h (n=1). **e**, Immunoblot analysis of HeLa cells treated with indicated esiRNAs (representative immunoblot from n=3 independent cultures. **f**, Nucleotide level in WT HeLa cells treated with indicated esiRNAs (n=5 independent cultures). **g**, ISG expression in HeLa cells treated with 40 µM ddC for 6 days and the indicated esiRNA (n=3 independent cultures). *P* values calculated using two-tailed unpaired t-test (**a,c,f**); two-way ANOVA with Tukey`s multiple comparison test (**b** and **g**). NS= not significant. Data are means ± SEM. Source data

Because nucleotide deficiencies drive ISG expression in *Yme1l*^*−*/*−*^ cells, this raised the intriguing possibility that an impaired nucleotide metabolism may induce a mtDNA-dependent inflammatory response also in the presence of YME1L. To mimic the effect of impaired glutamine utilization as observed in *Yme1l*^*−*/*−*^ cells, we inhibited glutaminolysis in WT cells using bis-2-(5-phenylacetamido-1,3,4-thiadiazol-2-yl) ethyl sulfide (BPTES), an inhibitor of glutaminase (GLS) that converts glutamine to glutamate. We observed diminished nucleotide levels in BPTES-treated cells (Extended Data Fig. 8a), which was accompanied by an increased cGAS- and STING-dependent ISG expression (Extended Data Fig. 8b). Treatment of cells with another GLS inhibitor, GLS968, also triggered ISG expression (Extended Data Fig. 8c). Depletion of mtDNA by ddC dampened the ISG response to GLS inhibition (Fig. 7a), demonstrating that inhibition of glutaminolysis in cultured cells is sufficient to reduce cellular nucleotide levels and activate cGAS–STING signalling in a mtDNA-dependent manner.

Our results in *Yme1l*^*−/−*^ cells indicated that depletion of pyrimidines specifically is sufficient to induce mtDNA-dependent ISG expression. We tested this further in WT cells and monitored ISG expression upon inhibition of de novo pyrimidine or purine synthesis. Although inhibition of purine synthesis by lometrexol or 6-mercaptopurine did not trigger ISG expression, we observed a mild innate immune response in cells treated with the dihydroorotate dehydrogenase inhibitor leflunomide and robust induction of ISG expression upon the inhibition of thymidylate synthase by 5-fluoruracil (5-FU) (Fig. 7b). Treatment with 5-FU caused the depletion of thymidine nucleotides (Fig. 7c), as expected. Supplementation of cells with thymidine alone completely suppressed ISG expression in response to 5-FU (Fig. 7d), whereas it did not inhibit cGAS activation in response to dsDNA transfection (Extended Data Fig. 8d). Furthermore, ISG expression was blocked in 5-FU-treated cells upon *cGas* knockdown (Fig. 7e). These results demonstrate that inhibition of thymidine synthase and depletion of thymidine nucleotides is sufficient to induce an innate immune response downstream of cGAS signalling.

Inhibition of pyrimidine synthesis can induce nuclear DNA damage^36^, which could conceivably trigger cGAS activation via nuclear DNA. To monitor the contribution of mtDNA to the 5-FU-induced immune response, we depleted mtDNA with ddC. Loss of mtDNA ameliorated ISG expression in 5-FU-treated cells, highlighting the contribution of mtDNA to the inflammatory response (Fig. 7f). Consistently, inhibition of VDAC oligomerization by VBIT-4 impaired the immune response upon thymidylate synthase inhibition (Fig. 7g).

We corroborated these findings by inhibiting de novo synthesis of pyrimidines genetically. Downregulation of the multifunctional biosynthetic enzyme carbamoyl-phosphate synthetase 2, aspartate transcarbamoylase and dihydroorotase (CAD) decreased pyrimidine levels in cells and broadly induced ISG expression (Fig. 7h and Extended Data Fig. 8e,f). Genome-wide expression profiling of CAD-depleted cells revealed that the increased expression of ~80% of ISGs was suppressed if cells were depleted of mtDNA by ddC treatment (Fig. 7h and Extended Data Fig. 8g).

Together, we conclude that impaired de novo nucleotide synthesis in the cytosol boosts the expression of ISGs, the majority of them in an mtDNA-dependent manner. Moreover, these experiments unravelled a striking specificity of the mtDNA-dependent innate immune response for disturbances in the synthesis of pyrimidines.

## Discussion

**Fig. 8:**
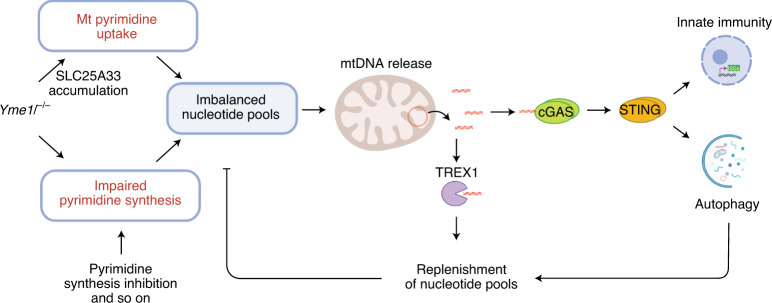
mtDNA-dependent innate immunity is coupled to cellular nucleotide metabolism. The *i*-AAA protease YME1L is required for efficient de novo pyrimidine synthesis and limits accumulation of the mitochondrial pyrimidine transporter SLC25A33. Loss of YME1L or cytosolic pyrimidine synthesis inhibition leads to an imbalance in cellular nucleotide pools. Deregulated mitochondrial nucleotide uptake and mtDNA replication may trigger the release of mtDNA into the cytosol. Cytosolic mtDNA is either degraded by the exonuclease TREX1 to replenish cytosolic nucleotide pools or binds to cGAS, which induces a STING-dependent innate immune response and autophagy.

Our results demonstrate that nucleotide metabolism orchestrates mtDNA-dependent innate immunity. We specifically identify the mitochondrial protease YME1L as a central regulatory node linking nucleotide homeostasis with mtDNA sensing by the cytosolic cGAS–STING–TBK1 pathway (Fig. 8). YME1L preserves cytosolic nucleotide pools by ensuring their synthesis from glutamine and degrades the pyrimidine carrier SLC25A33, thereby limiting the transport of pyrimidine nucleotides across the mitochondrial inner membrane. Genetic intervention and complementation studies reveal that, specifically, the depletion of pyrimidines in the cytosol drives an mtDNA-dependent immune response in *Yme1l*^*−*/*−*^ cells. Similarly, disturbances in the de novo synthesis of pyrimidines, but not of purines, are sufficient to trigger ISG expression by mtDNA in WT cells, highlighting the general importance of mtDNA for innate immune signalling if pyrimidine metabolism is impaired. mtDNA may therefore also contribute to the antiviral properties of pyrimidine inhibitors, which involve induction of a cellular immune response^37,38^.

Loss of YME1L induces the expression of a similar set of ISGs as observed in *Tfam*^+/*−*^ cells^4^. However, the mtDNA-dependent innate immune response in *Tfam*^+/*−*^ cells is independent of SLC25A33 and pyrimidine levels, indicating the presence of different signalling pathways that trigger the release of mtDNA. Although mtDNA moderately accumulates in *Yme1l*^*−*/*−*^ cells, decreased expression of TFAM in *Tfam*^+/*−*^ cells is associated with lower mtDNA levels^39^. We propose that, rather than altered mtDNA levels per se, an imbalanced mitochondrial nucleotide supply due to impaired de novo nucleotide synthesis or accumulation of SLC25A33 causes mtDNA stress and the release of mtDNA from mitochondria. Consistent with this model, increased mtDNA replication was found to deplete cytoplasmic nucleotide pools in induced pluripotent stem cells derived from mtDNA mutator mice^33^ and to induce inflammation in macrophages via activation of inflammasomes^40^.

In contrast to apoptotic cells, the accumulation of cytosolic mtDNA in YME1L- or TFAM-deficient cells does not depend on BAX/BAK pores in the outer membrane, but requires VDAC oligomerization, suggesting the release of mtDNA fragments^3^. Notably, yeast *YME1* (*Yeast Mitochondrial Escape 1*) was originally identified in a genetic screen for mutants facilitating transfer of mtDNA to the nucleus^41^, demonstrating that Yme1 suppresses mtDNA release also in an organism lacking an inflammatory response. It is conceivable that, independent of its function for immune signalling, cytoplasmic mtDNA replenishes nucleotide pools in the cytoplasm, either directly upon degradation by the cytosolic nuclease TREX1 or by stimulating autophagy, a primordial function of cGAS–STING signalling (Fig. 8)^42^. In agreement with this scenario, autophagy was found to balance synthesis and degradation of mtDNA in starved yeast cells^43^.

The discovery that cellular nucleotide deficiencies can cause mtDNA release and inflammation has broad pathophysiological implications. Targeting cellular nucleotide metabolism and mitochondrial nucleotide transport might offer new therapeutic avenues for inflammatory diseases linked to mtDNA, including heart and neurodegenerative diseases and obesity^44–46^. Furthermore, mtDNA-dependent inflammation must be considered in diseases associated with disturbed nucleotide metabolism such as neurological diseases and cancer. It is noteworthy that mutation of *YME1L* has also been associated with increased transfer of mtDNA to the nuclear genome in colorectal cancer^47^. We recently reported that YME1L promotes metabolic rewiring of mitochondria to facilitate the growth of pancreatic ductal adenocarcinoma cells^18^. Our current findings raise the intriguing possibility that YME1L inhibition may limit tumour growth both by preventing metabolic reprogramming of mitochondria and by promoting mtDNA-dependent inflammation and the production of type I interferons, which are known to induce senescence in numerous human cancers^48^.

## Methods

### Mouse breeding and tissue collection

Animal procedures complied with all relevant ethical regulations, were carried out in accordance with European, national and institutional guidelines and were approved by local authorities (Landesamt für Natur, Umwelt und Verbraucherschutz Nordrhein-Westfalen, Germany; approval number: 84-02.04.2014.A418.) Mice were maintained at the specific-pathogen-free animal facility of the CECAD Research Centre (ambient temperature 22 ± 2 °C, humidity 55 ± 10%) under a 12 h light cycle and were given a regular chow diet (catalogue no. Ssniff V1154-330).

Nervous-system-specific *Yme1l* knockout mice were as described previously^19^. Groups included male and female C57BL/6N animals at 6–7 or 31–32 weeks of age. Samples for protein and RNA extraction were taken after cervical dislocation and frozen in liquid nitrogen.

### Protein digestion for proteomics (retina)

Mouse retinas were lysed and in-solution digested with trypsin according to the method established in our facility using guanidinium chloride buffer^49^. Before loading to liquid chromatography–mass spectrometry (LC–MS), the resulting peptides were cleaned with C18 StageTips.

### Mass spectrometric analysis (LC–MS/MS) of the retina

The peptides were analysed using an Orbitrap Fusion mass spectrometer (Thermo Fisher Scientific) with a nano-electrospray ion source, coupled with an EASY-nLC 1000 (Thermo Fisher Scientific) UHPLC. A 25-cm-long reversed-phase C18 column with 75 μm inner diameter (PicoFrit, LC Packings) was used for separating peptides. The LC runs lasted 140 min with a concentration of 6% solvent B (0.1% formic acid in 80% acetonitrile) increasing to 31% over 120 min and further to 50% over 10 min. The column was subsequently washed and re-equilibrated. The flow rate was 200 nl min^−1^. MS spectra were acquired in a data-dependent manner with a top speed method. For mass spectrometry (MS), the mass range was set to 350–1800 *m*/*z* and resolution to 60 K at 200 *m*/*z*. The AGC target of MS was set to 1 × 10^6^, and the maximum injection time was 100 ms. Peptides were fragmented with higher-energy collisional dissociation with collision energy of 25. The resolution was set to 30 K. The AGC target of MS/MS was 2 × 10^5^ and the maximum injection time was 55 ms.

### Bioinformatics and data analysis proteomics (retina)

MaxQuant v.1.5.3.8 (ref. ^50^) with integrated Andromeda search engine^51^ was used for analysing the liquid chromatography tandem mass spectrometry (LC–MS/MS) raw data. The raw data were searched against the mouse proteome from UniProt (knowledgebase 2017_01). The following parameters were used for data analysis: for ‘fixed modification’, cysteine carbamidomethylation and methionine oxidation; for ‘variable modification’, methionine oxidation and protein N-terminal acetylation; for ‘digestion’ specific with Trypsin/P, maximum missed cleavages 2; for label-free quantification, match between runs is selected. Other parameters were set as default.

### Protein digestion for proteomics (MEFs)

MEFs were lysed in 1% sodium laurate by sonication and heating to 70 °C for 20 min. 50 µg protein lysate were used for digestion. Proteins were reduced and alkylated using 10 mM tris-(2-carboxethyl)-phosphine and 55 mM 2-chloroacetamide (45 min, room temperature in the dark), respectively. Lys-C (Wako) was added at a ratio of 1:100 (enzyme/protein) and incubated for 2 h at room temperature. The sodium laurate concentration was then diluted to 0.1% using 10 mM HEPES–KOH pH 8.5 and trypsin was added at a ratio of 1:100 (enzyme/protein). Digestion was stopped after overnight incubation at 37 °C, using trifluoric acid at a final concentration of 0.1%. Sodium laurate was removed by phase transfer method from samples after acidification and generated peptides were desalted using the SDB StageTip technique^52^.

### Mass spectrometric analysis (LC–MS/MS) of MEFs

LC–MS/MS instrumentation consisted of a nano-LC 1200 (Thermo Fisher Scientific) coupled via a nano-electrospray ionization source to a quadrupole-based Q Exactive HF-x instrument.

Peptides were separated on in-house packed 50-cm columns (1.8 µm C18 ReproSil Dr Maisch) within a total gradient length of 90 min. The column temperature was controlled by an in-house-designed column oven at 50 °C. The flow rate was 250 nl min^−1^. The spray settings were: 2.4 kV; capillary temperature, 275 °C; no auxiliary gas applied.

The mass spectrometer operated in a data-dependent mode targeting the top 22 intense peaks for fragmentation and MS/MS spectra acquisition. MS/MS spectra were acquired at 17,500 resolution (200 *m*/*z*) using an isolation window of 1.4 Th. The maximum injection time was set to 22 ms. The normalized collision energy was set to 29 and dynamic exclusion was enabled for 20 s.

### Bioinformatics and data analysis proteomics (MEFs)

Acquired raw data were analysed using MaxQuant v.1.5.3.8 and the implemented Andromeda search engine^50^. MS/MS spectra were compared with the Uniprot *Mus musculus* reference proteome database containing isoforms. Default settings for mass accuracy and search parameters were applied. The match between runs and maxLFQ algorithms were enabled^53^.

Data were further analysed and visualized using Instant Clue^54^. Differentially expressed proteins were identified by a two-sided *t*-test followed by a permutation based false discovery rate (FDR) estimation (FDR < 5%, s0 = 0.1)^55^.

### Whole-cell metabolite analysis

Cells were seeded one day before metabolite extraction. Cells were washed twice with ammonium carbonate (75 mM, pH 7.4) before two 10-min incubation steps with extraction buffer (40:40:20 v/v/v acetonitrile/methanol/water containing 10 ng ml^−1^ ^13^C_10_-ATP as an internal standard (Sigma, catalogue no. 710695)) at −20 °C. The cells were collected in the extraction buffer and centrifuged at 14,500*g* at 4 °C for 10 min. Supernatants were transferred to fresh tubes and dried down using a speed vac concentrator (Eppendorf). Samples were resuspended in 100 µl of LC–MS-grade H_2_O (Thermo Fisher Scientific) of which 80 µl were used to perform anion-exchange chromatography (ICS 5000 (Thermo Fisher Scientific)) as described previously^56^; 20 µl were used for the analysis of amino acids using a benzoylchloride derivatization method^57^.

Analysis of anionic metabolites in brief: 5 µl of the polar metabolite extract was injected on a Dionex IonPac AS11-HC column (2 mm × 250 mm, 4-μm particle size (Thermo Fisher Scientific)). Compounds were eluted using the following potassium hydroxide gradient at a flow rate of 380 µl min^−1^: 0–5 min, 10–25 mM KOH; 5–21 min, 25–35 mM KOH; 21–25 min, 35–100 mM KOH, 25–28 min, 100 mM KOH, 28–32 min, 100–10 mM KOH.

The eluting metabolites were detected in negative ion mode using multiple reaction monitoring (MRM) on a Waters TQ triple quadrupole mass spectrometer (Waters) or in full-scan mode on a Q Exactive HF (Thermo Fisher Scientific). The MS settings for the MRM analysis were: capillary voltage, 2.7 kV; desolvation temperature, 550 °C; desolvation gas flow, 800 l h^−1^; collision cell gas flow, 0.15 ml min^−1^. The MS settings for the high resolution, full-scan MS analysis were: spray voltage, −3.0 kV; capillary temperature, 300 °C; sheath gas flow, 60 AU; and aux gas flow, 20 AU at a temperature of 300 °C. The S-lens was set to a value of 60 AU.

All peaks on the triple quadrupole were validated using two MRM transitions one for quantification of the compound, whereas the second ion was used for qualification of the identity of the compound. The settings for the MRM transitions are given in Supplementary Table 1.

Data analysis and peak integration was performed using TargetLynx software (Waters). Data analysis on the Q Exactive was performed using TraceFinder software (Thermo Fisher Scientific). The identity of each compound was validated by reference compounds. Peak areas of [M – H]^*−*^ ions were extracted using a mass accuracy (<5 ppm) and a retention time tolerance of <0.2 min. Peak areas were normalized to the internal standard followed by total protein amounts, which were determined using a BCA assay kit (Thermo Fisher Scientific).

Analysis of amino acids in brief: 20 µl of the sample resuspended for the anion-exchange chromatography was mixed with 10 µl of 100 mM sodium carbonate (Sigma) followed by the addition of 10 µl of 2% benzoylchloride (Sigma) in acetonitrile (Optima-Grade, Thermo Fisher Scientific). Samples were vortexed before centrifugation for 10 min 21,300*g* at 20 °C.

For the analysis, 1 µl of the derivatized sample was injected onto a 100 × 2.1 mm HSS T3 UPLC column (Waters). The flow rate was set to 400 µl min^−1^ using a buffer system consisted of buffer A (10 mM ammonium formate (Sigma), 0.15% formic acid (Sigma) in Milli-Q water (Millipore)) and buffer B (acetonitrile, optima-grade (Thermo Fisher Scientific)). The LC gradient was: 0% B at 0 min; 0–15% B at 0–0.1 min; 15–17% B at 0.1–0.5 min; 17–55% B at 0.5–7 min; 55–70% B at 7–7.5 min; 70–100% B at 7.5–9 min; 100% B at 9–10 min; 100–0% B at 10–10.1 min; 0% B at 10.1–15 min. The mass spectrometer was operating in positive ionization mode monitoring the mass range *m*/*z* 50–750. The heated electrospray ionization source settings of the mass spectrometer were: spray voltage, 3.5 kV; capillary temperature, 250 °C; sheath gas flow, 60 AU; and aux gas flow, 20 AU at a temperature of 250 °C. The S-lens was set to a value of 60 AU.

Data analysis was performed using the TraceFinder software (Thermo Fisher Scientific). The identity of each compound was validated by reference compounds. Peak areas of [M + Bz + H]^+^ ions were extracted using a mass accuracy (<5 ppm) and a retention time tolerance of <0.2 min.

### Cell culture, transfection and RNA interference

HeLa cells and MEFs were maintained in DMEM-GlutaMAX (Life Technologies) containing 4.5 g l^−1^ of glucose supplemented with 1 mM sodium pyruvate (Gibco), 100 μM non-essential amino acids (Gibco) and 10% fetal bovine serum (Biochrom). Cell lines were maintained at 37 °C and 5% CO_2_ and were routinely tested for mycoplasma.

Cell numbers were monitored by Trypan blue exclusion and cell counting using the Countess automated cell counter (Thermo Fisher Scientific). Cells were seeded at equal densities and grown to confluency over a period of 72 h without medium changes unless stated otherwise. Opti-MEM + GlutaMAX (Gibco) and lipofectamine RNAiMax (Invitrogen) were used for reverse transfection of endoribonuclease-prepared short interfering RNA (esiRNA) and short interfering RNA (siRNA) for 72 h. The esiRNA and siRNAs used in this study can be found in Supplementary Table 2. Where indicated, the following compounds were added to the medium: BX795 (Sigma), 2′,3′-dideoxycytidine (ddC (Sigma)), VBIT-4 (Aobious), cycloheximide (Sigma), EtBr (Roth), bis-2-(5-phenylacetamido-1,3,4-thiadiazol-2-yl) ethyl sulfide (BPTES) (Sigma), compound 968 (GLS inhibitor 968 (Sigma)), 5-FU (Sigma), leflunomide (Enzo), 6-mercaptopurine (Sigma), lometrexol hydrate (Sigma), VACV-70 70 bp oligonucleotides (InvivoGen), poly(dA:dT) (InvivoGen) and HSV-60 (InvivoGen). For pyrimidine nucleoside supplementation, cells were cultured in cytidine, thymidine and uridine (each at 100 µM (Sigma)) for at least one passage before experiments.

### Generation of knockout and stable cell lines

Immortalized WT and *Yme1l*^*−/−*^ MEFs and HeLa cells were described previously^18^. In brief, primary MEFs were isolated from *Yme1l*^loxP/loxP^ animals and immortalized using SV40 large T antigen-encoding plasmids. Upon transduction with Cre recombinase, individual clones were expanded and genotyped to identify WT (*Yme1l*^loxP/loxP^) and *Yme1l*^*−*/*−*^ MEFs. *YME1L*^*−*/*−*^ HeLa cells and *Slc25a33*^*−*/*−*^ MEFs were generated using CRISPR–Cas9-mediated gene targeting. Guide RNA targeting *YME1L* (5′-GGAACCGACCATATTACAACAGG-3′) and Cas9 were expressed in HeLa cells using transient transfection of the px330 expression vector. Guide RNA targeting *Slc25a33* (5′-CGCGTCTTAATGACTTCTAG-3′) and Cas9 were expressed in WT and *Yme1l*^*−/−*^ MEFs using transient transfection of the px459 expression vector before puromycin selection. Individual clones were validated by immunoblotting and genomic sequencing. Unedited WT and *Yme1l*^*−*/*−*^ MEF clones were used as controls. HeLa cells expressing mouse SLC25A33–MycFlag were generated by lentiviral transduction and selected using 1 µg ml^−1^ puromycin. Lentiviral particles were generated by transfecting HEK293T cells with pLVXpuro–SLC25A33–MycFlag using Lenti-X Packaging Single Shots (Takara) and the viral supernatant was collected after 48 h. WT and *Yme1l*^*−/−*^ MEFs expressing HA-MITO were generated by retroviral transduction and selected using 10 µg ml^−1^ of blasticidin. Retrovirus was generated by transfecting HEK293T cells with pMXs–3XHA–EGFP–OMP25 (HA-MITO; a gift from David M. Sabatini, Addgene, catalogue no. 83356) using GeneJuice (Merck) and the viral supernatant was collected after 48 h. Primary MEFs were derived from WT and *Tfam*^*+/−*^ animals and immortalized by transfection with the SV40 large T antigen. Immortalized WT and *Bax*^*−/−*^*Bak*^*−/−*^ MEFs were a kind gift from Richard Youle (NINDS).

### Mitochondrial metabolite analysis

Relative metabolite levels were largely determined as described previously^31^. In brief, 30 × 10^6^ HA-MITO-expressing MEFs were plated one day before the experiment and all subsequent steps were performed at 4 °C. Cells were washed twice and collected in KPBS (136 mM KCl, 10 mM KH_2_PO_4_–KOH pH 7.25) before centrifugation at 1,000*g* for 2 min. Cells were resuspended in KPBS and homogenized with 15 strokes using a glass Teflon homogenizer at 1000 rpm. The homogenates were centrifuged at 1,000*g* for 3 min and the supernatant was incubated with magnetic anti-HA beads (Thermo Fisher Scientific) on an end-over-end rotator for 3 min at 15 r.p.m. Beads were collected on a magnet and washed four times with KPBS. Mitochondrial metabolites were extracted by incubating the beads with extraction buffer (40:40:20 v/v/v acetonitrile/methanol/water containing 10 ng ml^−1^ of ^13^C_10_-ATP (Sigma) as an internal standard) for 5 min. For each experiment, an input and IP fraction were taken for immunoblot analysis of mitochondrial enrichment and purity. The organellar-specific anionic metabolites were analysed as described in the whole-cell metabolite section. Data was normalized to the succinate dehydrogenase complex flavoprotein subunit A (SDHA) protein level determined for each IP fraction by immunoblot densitometry.

### Stable iotope enrichment analysis using ^13^C_5_^15^N_2_-glutamine

MEFs were grown and extracted as described in the whole-cell metabolite analysis section. Cells were washed twice with 1 ml of Dulbecco’s PBS (Gibco, catalogue no. 14190250) before adding the tracing buffer (DMEM + 10% dialysed FBS, 25 mM glucose, 1 mM sodium pyruvate and 2 mM l-glutamine-^13^C_5_^1^^5^N_2_ (Sigma, catalogue no. 607983)).

Metabolic analysis was performed as described previously^31^ using an Acquity iClass UPLC (Waters) equipped with a ZIC-pHILIC 2.1 × 150 mm (5-μm particle size) column (Sequant) coupled to an Q Exactive HF mass spectrometer (Thermo Fisher Scientific). The HPLC flow rate and gradient settings as well as MS settings were identical to those reported previously^31^.

Data analysis was performed using TraceFinder software (Thermo Fisher Scientific). The identity of each compound was validated by reference compounds and peak areas of selected ^13^C ^15^N or ^13^C^15^N isotopes of each analysed compound was extracted from either [M – H^+^]^*−*^ (nucleotides, α-ketoglutarate, succinate, fumarate, malate and citrate) or [M + H^+^]^+^ (glutamine, glutamate and aspartate) ions. For the differential isotope enrichment analysis, the sum of all peak areas of the extracted isotopes of a compound was calculated together with the fraction of the individual isotope. In addition, we calculated the atom fraction enrichment factor, which describes the relative contribution of stable isotopes to the total area of each compound. Isotope peaks were extracted using a mass accuracy (<5 ppm) and a retention time tolerance of <0.1 min. A list of analysed isotopologues and the individual metabolite pool sizes according to the sum of all isotopes are presented in Supplementary Table 1.

### RNA sequencing

Total RNA was extracted from cells using NucleoSpin RNA (Macherey–Nagel). Libraries were prepared using the Illumina TruSeq mRNA stranded sample preparation kit. Library preparation started with 1 μg of total RNA. After poly(A) selection (using poly(T) oligo-attached magnetic beads), mRNA was purified and fragmented using divalent cations under elevated temperature. The RNA fragments underwent reverse transcription using random primers. This was followed by second-strand cDNA synthesis with DNA Polymerase I and RNase H.

After end repair and A-tailing, indexing adaptors were ligated. The products were then purified and amplified (14 PCR cycles) to create the final cDNA libraries. After library validation and quantification (Agilent tape station), equimolar amounts of library were pooled. The pool was quantified by using the Peqlab KAPA Library Quantification Kit and the Applied Biosystems 7900HT Sequence Detection System. The pool was sequenced with a paired-end 100-nucleotide protocol on an Illumina NovaSeq6000 sequencer. For bioinformatic analysis of the Scr versus Yme1l siRNA and WT versus *Yme1l*^*−*/*−*^ MEF datasets, raw reads were mapped to mm10 (Ensembl build 91) with HiSat v.2.0.4 (ref. ^58^). Transcript assembly was performed using StringTie v.1.3.3 (ref. ^59^) and differential gene expression analysis was done with Cufflinks v.2.2.1 (ref. ^60^). For bioinformatic analysis of the HeLa cell SLC25A33 overexpression and CAD depletion datasets, raw reads were mapped to hg38 (Ensembl build 95) using kallisto-quant v.0.45.0. Differential gene expression was analysed using DESeq2 v.1.22.2. Statistical significance was determined using the Benjamini–Hochberg method. *P* values <0.05 were considered to be significant. The analysis of ddC-dependent ISG expression in HeLa cells overexpressing SLC25A33 (Fig. 2i) or depleted of CAD (Fig. 4h) was performed as follows. Significantly upregulated genes in SLC25A33–MycFlag or *CAD* esiRNA cells (log_2_(fold change) >1) compared with their respective controls were entered into the Interferome database^22^ to determine the number of ISGs upregulated in each condition in the absence of ddC. Next, the log_2_(fold change) of these ISGs was also determined in the presence of ddC in SLC25A33–MycFlag or *CAD* esiRNA compared with their respective controls. A minor fraction of these ISGs were differentially regulated by ddC treatment alone in WT or *Gfp* esiRNA control HeLa cells (log_2_(fold change) >1) and were therefore excluded.

### Quantitative PCR with reverse transcription

Retinas were homogenized and total RNA was isolated using QIAzol (Qiagen) and chloroform followed by purification using the NucleoSpin RNA isolation kit (Macherey–Nagel) according to the manufacturer’s protocol. Total RNA from cells was isolated upon cell lysis using the NucleoSpin RNA isolation kit. cDNA was synthesized using the GoScript Reverse Transcription Mix (Promega) and RT–qPCR was performed using PowerSYBR Green PCR Master Mix (Applied Biosystems). For each independent sample, RT–qPCR was performed in technical duplicates or triplicates. The primer sequences used in this study can be found in Supplementary Table 3.

### SDS–PAGE and immunoblot analysis

Cells were washed with cold PBS and resuspended in ice-cold RIPA buffer (50 mM Tris–HCl, pH 7.4, 150 mM NaCl, 1% Triton X-100, 0.1% SDS, 0.05% sodium deoxycholate, 1 mM EDTA) containing protease inhibitor cocktail (Roche) and phosphatase inhibitor cocktail (PhosSTOP, Roche). Lysates were incubated with constant agitation for 30 min at 4 °C followed by centrifugation at 14,500*g* for 10 min. Total protein (50–100 µg) was separated using SDS–PAGE, followed by transfer to nitrocellulose membranes and immunoblotting with the antibodies listed in Supplementary Table 4.

### Mitochondrial DNA isolation

mtDNA was isolated from cultured cells using the DNeasy Blood & Tissue Kit (Qiagen) according to the manufacturer’s instructions and quantitative polymerase chain reaction (qPCR) was performed using nuclear DNA (*βactin*) and mtDNA primers (*Cytb*, *Dloop*). For each independent sample, qPCR was performed in technical duplicates or triplicates.

### Detection of mtDNA in cytosolic extracts

MEF and HeLa cytosolic extracts were generated as described previously^4^. In brief, cells were collected at 600*g* for 3 min and resuspended in 500 µl of buffer containing 150 mM NaCl, 50 mM HEPES–KOH pH 7.4, and 15 µg ml^−1^ (MEFs) or 20 µg ml^−1^ (HeLa) digitonin (Calbiochem). The homogenates were incubated end-over-end for 10 min and centrifuged at 980*g* for 3 min. The first pellet was taken as the ‘Pellet’ fraction for immunoblotting. Centrifugation was repeated three times to clear the supernatant of intact cells. The cytosolic fractions were spun down at 17,000*g* for 10 min and DNA was isolated from the supernatant using the DNeasy Blood & Tissue Kit (Qiagen). qPCR was performed on cytosolic fractions using mtDNA primers (*Cytb*, *Dloop*). We detected very minor nuclear DNA in cytosolic fractions using nuclear DNA primers (*hTert or βactin* in MEFs and *βactin* in HeLa cells), which was used for normalization to control for any differences in membrane rupture between genotypes.

### Immunofluorescence

Cells were fixed on coverslips using growth medium containing 3.7% PFA for 15 min at room temperature. After PBS washes, cells were permeabilized using 0.1% Triton X-100 in PBS for 5 min and washed with PBS again. Coverslips were incubated with primary antibodies (Supplementary Table 3) for 1 h and washed three times with PBS before incubation with secondary antibodies (AlexaFluor (Invitrogen)) for 45 min in the dark. Upon incubation, cells were washed with PBS, stained with 4,6-diamidino-2-phenylindole, washed again with PBS and mounted using ProLong Gold (Invitrogen). Images were acquired using a Leica SP8-DLS laser-scanning confocal microscope equipped with an ×100 oil HC PL APO CS2 objective (numerical aperture (NA) 1.4) or Leica DMI 6000 B wide-field fluorescence microscope equipped with an ×100 oil HCX PL APO CS 23°–37 °C objective (NA 1.46).

### Enzyme-linked immunosorbent assay

The VeriKine Mouse IFN-β enzyme-linked immunosorbent assay (pbl assay science 42400-1) was carried out on cell culture supernatants of MEFs after 72 h in culture according to manufacturer’s instructions.

### Cell proliferation assay

Some 10,000 MEFs were seeded per well in a 96-well plate using the IncuCyte S3 Live-Cell Analysis System (Sartorius). Pictures were acquired every 2 h until confluency was reached for both genotypes.

### Statistics and reproducibility

Sample size was chosen according to our previous experience and common standards. No statistical method was used to predetermine sample size. The sample size included at least three independent cell cultures or mice where statistical evaluation was performed. We have not excluded any samples. Experiments were repeated as detailed in the figure legends. Mice were assigned to experimental groups based on genotypes. Analyses were not blinded because experiments were performed and analysed by the same researcher. The N number for all MEF or HeLa cell experiments represent independent experimental cell cultures. Data analysis was performed with Prism GraphPad 8 and Instant Clue. Images were processed with ImageJ National Institutes of Health and schematics were created with BioRender.com.

### Reporting Summary

Further information on the research design is available in the Nature Research Reporting Summary linked to this article.

## Supplementary information

Supplementary Information Supplementary Tables 2–4

Reporting Summary

Supplementary Table 1 Metabolomics data

## Data Availability

All data and materials are available from the corresponding author upon reasonable request. Proteomics from WT versus *Yme1l*^*−*/*−*^ MEFs and WT versus NYKO retinas have been deposited to the ProteomeXchange Consortium via the PRIDE partner repository and are accessed via their respective dataset identifiers; PXD018097 and PXD019849. All transcriptomic data have been deposited to the GEO omnibus under the SuperSeries accession number GSE161736, which contains the following RNA-Seq experiments: SLC25A33 overexpressing HeLa cells in the presence and absence of ddC (GSE161732); HeLa cells depleted of CAD in the presence and absence of ddC (GSE161733); MEFs treated with scrambled or *Yme1l* siRNA (GSE161734); WT versus *Yme1l*^*−/−*^ MEFs (GSE161735). Uniprot database is accessible via https://www.uniprot.org and the Interferome database via http://www.interferome.org. Source data are provided with this paper.

## Source data

Source Data Fig. 1 Unprocessed immunoblots. Source Data Fig. 3 Unprocessed immunoblots. Source Data Fig. 4 Unprocessed immunoblots. Source Data Fig. 5 Unprocessed immunoblots. Source Data Extended Data Fig. 2 Unprocessed immunoblots. Source Data Extended Data Fig. 3 Unprocessed immunoblots. Source Data Extended Data Fig. 4 Unprocessed immunoblots. Source Data Extended Data Fig. 7 Unprocessed immunoblots. Source Data Extended Data Fig. 8 Unprocessed immunoblots.

## Extended data

are available for this paper at 10.1038/s42255-021-00385-9.
