# Supplementary material for: Cellular pyrimidine imbalance triggers mitochondrial DNA–dependent innate immunity
Source: Nat Metab. 2021 Apr 26;3(5):636–50. doi: 10.1038/s42255-021-00385-9 (PMC8144018; doi:10.1038/s42255-021-00385-9)
Supplement: Supplementary file 1 — Supplementary Tables 2–4 [file 42255_2021_385_MOESM1_ESM.pdf]

nature portfolio

https://doi.org/10.1038/s42255-021-00385-9

Supplementary information

Cellular pyrimidine imbalance triggers mitochondrial DNA–dependent innate immunity

In the format provided by the authors and unedited

Supplementary Information

Cellular pyrimidine imbalance triggers mitochondrial DNA-dependent innate immunity

Hans-Georg Sprenger[1,2,†], Thomas MacVicar[1,†], Amir Bahat[1], Kai Uwe Fiedler[1], Steffen Hermans[1], Denise Ehrentraut[1], Katharina Ried[1], Dusanka Milenkovic[1], Nina Bonekamp[1], Nils-Göran Larsson[1,3], Hendrik Nolte[1], Patrick Giavalisco[1], and Thomas Langer[1,2,*]

[1]Max Planck Institute for Biology of Ageing, Cologne, Germany

[2]Cologne Excellence Cluster on Cellular Stress Responses in Aging-Associated Diseases (CECAD), University of Cologne, Cologne, Germany

[3]Department of Medical Biochemistry and Biophysics, Karolinska Institutet, Stockholm, Sweden

†These authors contributed equally

Correspondence to: TLanger@age.mpg.de

Supplementary Table 1: Metabolomics data.

Separate Excel file

Supplementary Table 2: esiRNAs / siRNAs used.

**Human Gene / Target**	**ID**	**Company**	**Type**
Gfp	EHUEGFP	Sigma	esiRNA
SLC25A33	EHU160961	Sigma	esiRNA
CAD	EHU130441	Sigma	esiRNA

**Mouse Gene / Target**	**ID**	**Company**	**Type**
Scrambled (Scr, negative control)	SIC001-10nmol	Sigma	siRNA
cGas	SASI_Mm01_00129826	Sigma	siRNA
Sting	SASI_Mm02_00424289	Sigma	siRNA
Slc25a33 #1	SASI_Mm02_00332248	Sigma	siRNA
Slc25a33 #2	MSS230288	Invitrogen	siRNA
Slc25a33 #3	MSS230289	Invitrogen	siRNA
Slc25a16#1	SASI_Mm01_00197283	Sigma	siRNA
Slc25a16#2	SASI_Mm01_00197284	Sigma	siRMA
Slc25a28#1	SASI_Mm01_00024682	Sigma	siRNA
Slc25a28#2	SASI_Mm01_00024683	Sigma	siRNA
Slc25a36#1	SASI_Mm01_00141849	Sigma	siRNA
Slc25a36#2	SASI_Mm01_00141850	Sigma	siRNA
Slc25a38#1	SASI_Mm01_00130641	Sigma	siRNA
Slc25a38#2	SASI_Mm01_00130642	Sigma	siRNA
Ass1	SASI_Mm01_00153169	Sigma	siRNA
Samhd1	SASI_Mm01_00193980	Sigma	siRNA
Mda5	SASI_Mm02_00332785	Sigma	siRNA
Mavs	SASI_Mm01_00042222	Sigma	siRNA
Rig-I	SASI_Mm01_00145086	Sigma	siRNA
Drp1	SASI_Mm01_00125369	Sigma	siRNA
Scrambled (Scr, negative control)	Medium GC Duplex	Invitrogen	siRNA
Yme1l	MSS219539	Invitrogen	siRNA
Trex1	EMU031351	Sigma	esiRNA
cGas	EMU074131	Sigma	esiRNA

Supplementary Table 3: SYBR green qRT-PCR primers used.

Human Gene	Primer	Sequence
HPRT	forward	TGACACTGGCAAAACAATGCA
	reverse	GGTCCTTTTCACCAGCAAGCT
IFI44	forward	CAGAATGGAAACTAGGACTATG
	reverse	TCTTCGCATCGAAAAACTTC
ISG15	forward	AGATCACCCAGAAGATCG
	reverse	TGTTATTCCTCACCAGGATGT
IFIT2	forward	ACCATGAGTGAGAACAAGAAT
	reverse	TTAGATAGGCCAGTAGGTTG
CAD	forward	TAGTCCTTGGCTCTGGCGTCTA
	reverse	TAGTCGGTGCTGACTGTCTCTG
CCCR2	forward	CAGGTGACAGAGACTCTTGGGA
	reverse	GGCAATCCTACAGCCAAGAGCT
DDIT4L	forward	CTATCACCCAGAGAGCCTGCTA
	reverse	GGACAGACAGTTCTCCAGCATTT
IGFBP5	forward	CGTGCTGTGTACCTGCCCAATT
	reverse	ACTTGTCCACGCACCAGCAGAT
nuclearActin	forward	AACAGACTCCCCATCCCAAG
	reverse	CCAGAGGCGTACAGGGATAG
mtDloop	forward	GTCCCTTGACCACCATCCTC
	reverse	GTAGCACTCTTGTGCGGGAT
mtCYTB	forward	AGACAGTCCCACCCTCACAC
	reverse	GGTGATTCCTAGGGGGTTGT

Mouse Gene	Primer	Sequence
Cgas	forward	GTGAGGACCAATCTAAGACGAG
	reverse	AGCATGTTTTCTCTATCCCGTG
Mda5	forward	ACAGTGAAAGAGAATCTTGG
	reverse	CTGCTCATAATGTTGGGTTC
Trex1	forward	ATCTTCTTAGACCTGGAAGC
	reverse	CCTGAGAAATGGAAGTGTTC
Zbp1	forward	TGTTGACTTGAGCACAGGAG
	reverse	TTCAGGCGGTAAAGGACTTG
Mx1	forward	GACCATAGGGGTCTTGACCAA
	reverse	AGACTTGCTCTTTCTGAAAAGCC
Isg15	forward	CTAGAGCTAGAGCCTGCAG
	reverse	AGTTAGTCACGGACACCAG
Ifi44	forward	CTGATTACAAAAGAAGACATGACAGAC
	reverse	AGGCAAAACCAAAGACTCCA
Ifi47	forward	GAACAGAAAGCCAAACCCATAG
	reverse	GAAATCAAACGCACCCAGATC
Usp18	forward	GAGAGGACCATGAAGAGGA
	reverse	TAAACCAACCAGACCATGAG
Yme1l	forward	CCAGTTTTCATACAGTCTCGGG
	reverse	CTCAAGATCTGTTCCTCTGTCC

Hprt	forward	TCCTCCTCAGACCGCTTTT
	reverse	CATAACCTGGTTCATCATCGC
Rig-I	forward	TTGAAAGACTTGGGTACAAC
	reverse	TCATCAGAGTGAAGACAGAC
Mavs	forward	CAGTAGAGAATTCAGAGCAAC
	reverse	TTAGGAGAGGGTATCAAAGAG
Bak	forward	TGATATTAACCGGCGCTACG
	reverse	AGCTGATGCCACTCTTAAATAGG
Ass1	forward	CGAAATGATCTGATGGAGTATG
	reverse	CTTGATTCTTGGGGTTTTCC
Samhd1	forward	AAGTGGAGTACAAGGTCAAG
	reverse	GATTTCCAACCTCCTTTTCTC
Sting	forward	CTCATTGTCTACCAAGAACC
	reverse	TAACCTCCTCCTTTTCTTCC
Bax	forward	CCTTTTTGCTACAGGGTTTC
	reverse	ATATTGCTGTCCAGTTCATC
Cmpk2	forward	AGTACTTGACCTAGTTGACC
	reverse	CATCATCAAAGATCTTCCTCC
Slc25a33	forward	CCTACAGGTTCTGAAATCAATC
	reverse	CAGTATTGCTATTAGGCACG
Slc25a16	forward	ACCCCAATGTCTTAGTTTTG
	reverse	AATGGGTAGGATATCGTCTG
Slc25a28	forward	CATTGAGTGACGTAATCCAC
	reverse	TGCATCATGAAGTAATGTCG
Slc25a36	forward	CTCCCTCCAGAGCAATATAC
	reverse	TCTTTATAAGCCAAATGGGG
Slc25a38	forward	CAGACGTGATCAAAACTCAC
	reverse	CCATGGTTCTTGAAGATGAG
mtCytb	Mouse mtDNA CytB F	GCTTTCCACTTCATCTTACCATTTA
	Mouse mtDNA CytB R	TGTTGGGTTGTTTGATCCTG
nuclearActin	nDNA mouse ActB F	CATTGCTGACAGGATGCAGAAGG
	nDNA mouse ActB R	TGCTGGAAGGTGGACAGTGAGG
Dloop1	forward	AATCTACCATCCTCCGTGAAACC
	reverse	TCAGTTTAGCTACCCCCAAGTTTAA
Dloop2	forward	CCCTTCCCATTTGGTCT
	reverse	TGGTTTCACGGAGGATGG
Dloop3	forward	TCCTCCGTGAAACCAACAA
	reverse	AGCGAGAAGAGGGGCATT

Supplementary Table 4: Antibodies used.

Protein	Species	Company	Identifier
STAT1	rabbit	Cell Signaling Technology	Cat#9172 (1:1000)
RIG-I	rabbit	Cell Signaling Technology	Cat#3743 (1:500)
STING	rabbit	Cell Signaling Technology	Cat#13647 (1:1000)
YME1L	rabbit	ProteinTech	Cat#11510-1-AP (1:1000)
ACTIN	mouse	Sigma-Aldrich	Cat#A5441 (1:5000)
cGAS	rabbit	Cell Signaling Technology	Cat#31659 (1:500)
GAPDH	mouse	Santa Cruz Biotechnology Inc.	Cat#sc-32233 (1:1000)
SDHA	mouse	Invitrogen	Cat#459200 (1:2000)
Calnexin	rabbit	Sigma-Aldrich	Cat#208880 (1:1000)
SLC25A33	rabbit	Origene	Cat#TA309042 (1:1500)
γH2AX Ser139	mouse	Cell Signaling Technology	Cat#80312 (1:1000)
TOMM20	rabbit	Sigma-Aldrich	Cat#HPA011562 (1:2000 WB; 1:500 IF))
Citrate Synthase	rabbit	ProteinTech	Cat#16131-1-AP (1:5000)
ATP6V1A	rabbit	GeneTex	Cat#GTX110815 (1:1000)
LAMINB1	rabbit	Abcam	Cat#ab16048 (1:750)
BAK	rabbit	Sigma-Aldrich	Cat#06-536 (1:500)
BAX	rabbit	Cell Signaling Technology	Cat#2772 (1:500)
CAD	rabbit	Abcam	Cat#ab40800 (1:1000)
CHK1	mouse	Cell Signaling Technology	Cat#2360 (1:1000)
pCHK1 Ser345	rabbit	Cell Signaling Technology	Cat#2348 (1:1000)
DNA	mouse	Sigma-Aldrich	Cat#CBL186 (1:1000)
ATP5B	mouse	Invitrogen	Cat#A21351 (1:500)
