# Supplementary material for: Cellular pyrimidine imbalance triggers mitochondrial DNA–dependent innate immunity
Source: Nat Metab. 2021 Apr 26;3(5):636–50. doi: 10.1038/s42255-021-00385-9 (PMC8144018; doi:10.1038/s42255-021-00385-9)
Supplement: Supplementary file 2 — Reporting Summary [file 42255_2021_385_MOESM2_ESM.pdf]

nature research

Corresponding author(s): Thomas Langer

Last updated by author(s): Feb 27, 2021

Reporting Summary

Nature Research wishes to improve the reproducibility of the work that we publish. This form provides structure for consistency and transparency in reporting. For further information on Nature Research policies, see our Editorial Policies and the Editorial Policy Checklist.

Statistics

For all statistical analyses, confirm that the following items are present in the figure legend, table legend, main text, or Methods section.

n/a	Confirmed	
☐	☒	The exact sample size (*n*) for each experimental group/condition, given as a discrete number and unit of measurement
☐	☒	A statement on whether measurements were taken from distinct samples or whether the same sample was measured repeatedly
☐	☒	The statistical test(s) used AND whether they are one- or two-sided<br>*Only common tests should be described solely by name; describe more complex techniques in the Methods section.*
☒	☐	A description of all covariates tested
☐	☒	A description of any assumptions or corrections, such as tests of normality and adjustment for multiple comparisons
☐	☒	A full description of the statistical parameters including central tendency (e.g. means) or other basic estimates (e.g. regression coefficient) AND variation (e.g. standard deviation) or associated estimates of uncertainty (e.g. confidence intervals)
☐	☒	For null hypothesis testing, the test statistic (e.g. *F*, *t*, *r*) with confidence intervals, effect sizes, degrees of freedom and *P* value noted<br>*Give P values as exact values whenever suitable.*
☒	☐	For Bayesian analysis, information on the choice of priors and Markov chain Monte Carlo settings
☒	☐	For hierarchical and complex designs, identification of the appropriate level for tests and full reporting of outcomes
☒	☐	Estimates of effect sizes (e.g. Cohen's *d*, Pearson's *r*), indicating how they were calculated

Our web collection on statistics for biologists contains articles on many of the points above.

Software and code

Policy information about availability of computer code

Data collection	No software was used
Data analysis	Prism GraphPad 8 Software RRID:SCR_002798 Version 8.20<br>ImageJ National Institutes of Health 1.48u; RRID:SCR_003070<br>InstantClue Nolte et al., 2018 http://www.instantclue.uni-koeln.de/ Version 0.5.3<br>Waters MassLynx (TargetLynx Xs) Version 4.1<br>Thermo Fisher Scientific TraceFinder Version 4.1

For manuscripts utilizing custom algorithms or software that are central to the research but not yet described in published literature, software must be made available to editors and reviewers. We strongly encourage code deposition in a community repository (e.g. GitHub). See the Nature Research guidelines for submitting code & software for further information.

Data

Policy information about availability of data

All manuscripts must include a data availability statement. This statement should provide the following information, where applicable:

- Accession codes, unique identifiers, or web links for publicly available datasets
- A list of figures that have associated raw data
- A description of any restrictions on data availability

All data and materials are available from the corresponding author upon reasonable request. Proteomics from WT versus Yme1l-/- MEFs and WT vs NYKO retinas have been deposited to the ProteomeXchange Consortium via the PRIDE partner repository and are accessed via their respective dataset identifiers; PXD018097 and PXD019849. All transcriptomic data have been deposited to the GEO omnibus under the SuperSeries accession number GSE161736, which contains the following RNA-seq experiments: SLC25A33 overexpressing HeLa cells in the presence and absence of ddC (GSE161732); HeLa cells depleted of CAD in the presence

and absence of ddC (GSE161733); MEFs treated with scrambled or Yme1l siRNA (GSE161734); WT versus Yme1l-/- MEFs (GSE161735). Uniprot database is accessible via https://www.uniprot.org and the Interferome database via http://www.interferome.org.

Field-specific reporting

Please select the one below that is the best fit for your research. If you are not sure, read the appropriate sections before making your selection.

☒ Life sciences ☐ Behavioural & social sciences ☐ Ecological, evolutionary & environmental sciences

For a reference copy of the document with all sections, see nature.com/documents/nr-reporting-summary-flat.pdf

Life sciences study design

All studies must disclose on these points even when the disclosure is negative.

Sample size	Sample size was chosen according to our previous experience and common standards in the field (PMID: 31695197, 30389680). No statistical method was used to predetermine sample size. The sample size included at least 3 biological replicates where statistical evaluation was performed.
Data exclusions	We have not excluded any samples.
Replication	Experiments were repeated as detailed in the figure legends. All attempts at replication were successful. In some cases, multiple cell lines and additional siRNA sequences were used to verify the reproducibility of the findings.
Randomization	Mice were assigned to experimental groups based on genotypes. Cells were allocated by genotype and treatment, e.g. cells treated with the same chemical or siRNA were allocated to the same group.
Blinding	Analyses were not blinded because experiments were performed and analyzed by the same researchers.

Reporting for specific materials, systems and methods

We require information from authors about some types of materials, experimental systems and methods used in many studies. Here, indicate whether each material, system or method listed is relevant to your study. If you are not sure if a list item applies to your research, read the appropriate section before selecting a response.

Materials & experimental systems

n/a	Involved in the study	
☐	☒	Antibodies
☐	☒	Eukaryotic cell lines
☒	☐	Palaeontology and archaeology
☐	☒	Animals and other organisms
☒	☐	Human research participants
☒	☐	Clinical data
☒	☐	Dual use research of concern

Methods

n/a	Involved in the study	
☒	☐	ChIP-seq
☒	☐	Flow cytometry
☒	☐	MRI-based neuroimaging

Antibodies

Antibodies used

STAT1 rabbit Cell Signaling Technology Cat#9172
RIG-I rabbit Cell Signaling Technology Cat#3743
STING rabbit Cell Signaling Technology Cat#13647
YME1L rabbit ProteinTech Cat#11510-1-AP
ACTIN mouse Sigma-Aldrich Cat#A5441
cGAS rabbit Cell Signaling Technology Cat#31659
GAPDH mouse Santa Cruz Biotechnology Inc. Cat#sc-32233
SDHA mouse Invitrogen Cat#459200
Calnexin rabbit Sigma-Aldrich Cat#208880
SLC25A33 rabbit Origene Cat#TA309042
γH2AX Ser139 mouse Cell Signaling Technology Cat#80312
TOMM20 rabbit Sigma-Aldrich Cat#HPA011562
Citrate Synthase rabbit ProteinTech Cat#16131-1-AP
ATP6V1A rabbit GeneTex Cat#GTX110815
LAMINB1 rabbit Abcam Cat#ab16048
BAK rabbit Sigma-Aldrich Cat#06-536
BAX rabbit Cell Signaling Technology Cat#2772
CAD rabbit Abcam Cat#ab40800

	CHK1 mouse Cell Signaling Technology Cat#2360<br>pCHK1 Ser345 rabbit Cell Signaling Technology Cat#2348<br>DNA mouse Sigma-Aldrich Cat#CBL186<br>ATP5B mouse Invitrogen Cat#A21351<br>Alexa Fluor-568 anti-rabbit Invitrogen #A11011<br>Alexa Fluor-488 anti-mouse Invitrogen #A11001
Validation	Validation for commercially available antibodies can be found using the links below:<br>STAT1 https://www.cellsignal.de/products/primary-antibodies/stat1-antibody/9172<br>RIG-I https://www.cellsignal.de/products/primary-antibodies/rig-i-d14g6-rabbit-mab/3743<br>STING https://www.cellsignal.de/products/primary-antibodies/sting-d2p2f-rabbit-mab/13647<br>YME1L https://www.ptglab.com/products/YME1L1-Antibody-11510-1-AP.htm<br>ACTIN https://www.sigmaaldrich.com/catalog/product/sigma/a5441<br>cGAS https://www.cellsignal.de/products/primary-antibodies/cgas-d3o8o-rabbit-mab-mouse-specific/31659<br>GAPDH https://datasheets.scbt.com/sc-32233.pdf<br>Calnexin https://www.sigmaaldrich.com/catalog/product/mm/208880?lang=de&region=DE<br><br>SLC25A33 https://cdn.origene.com/datasheet/ta309042.pdf. We validated this antibody further in the lab using Slc25a33 knockdown and overexpression cell lines.<br><br>γH2AX Ser139 https://www.cellsignal.com/products/primary-antibodies/phospho-histone-h2a-x-ser139-d7t2v-mouse-mab/80312<br>TOMM20 https://www.sigmaaldrich.com/catalog/product/sigma/hpa011562<br>Citrate Synthase https://www.ptglab.com/Products/Pictures/pdf/16131-1-AP.pdf<br>ATP6V1A https://www.genetex.com/PDF/Download?catno=GTX110815<br>LAMINB1 https://www.abcam.com/lamin-b1-antibody-nuclear-envelope-marker-ab16048.pdf<br>BAK https://www.sigmaaldrich.com/catalog/product/mm/06536?<br>BAX https://www.cellsignal.de/products/primary-antibodies/bax-antibody/2772<br>CAD https://www.abcam.com/cadbm1-antibody-ep710y-ab40800.html<br>CHK1 https://www.cellsignal.de/products/primary-antibodies/chk1-2g1d5-mouse-mab/2360<br>p-CHK1 https://www.cellsignal.de/products/primary-antibodies/phospho-chk1-ser345-133d3-rabbit-mab/2348<br>DNA https://www.sigmaaldrich.com/catalog/product/mm/cbl186?lang=de&region=DE<br>ATP5B https://www.thermofisher.com/order/genome-database/dataSheetPdf?<br>producttype=antibody&productsubtype=antibody_primary&productId=A-21351&version=105

Eukaryotic cell lines

Policy information about cell lines

Cell line source(s)	HeLa (CCL-2) cells were purchased from ATCC. MEFs were described in PMID: 24616225. HEK293T cells were described in PMID: 31695197 but only used for virus production and no biological interpretations were based on HEK293T cells. Further cell line details are included in the Methods section.
Authentication	None of the cell lines were authenticated.
Mycoplasma contamination	All cell lines were routinely tested for mycoplasma by PCR. All cell lines were negative.
Commonly misidentified lines<br>(See ICLAC register)	No commonly misidentified cell lines were used.

Animals and other organisms

Policy information about studies involving animals; ARRIVE guidelines recommended for reporting animal research

Laboratory animals	C57BL/6N mice, male and female 6-7 or 31-32 week old mice
Wild animals	No wild animals.
Field-collected samples	No samples collected from the field.
Ethics oversight	All animal procedures were carried out in accordance with European, national and institutional guidelines and were approved by local authorities (Landesamt für Natur, Umwelt, und Verbraucherschutz Nordrhein-Westfalen, Germany; approval number: 84-02.04.2014.A418.

Note that full information on the approval of the study protocol must also be provided in the manuscript.
