# Supplementary figures and images for: Cellular pyrimidine imbalance triggers mitochondrial DNA–dependent innate immunity
Source: Nat Metab. 2021 Apr 26;3(5):636–50. doi: 10.1038/s42255-021-00385-9 (PMC8144018; doi:10.1038/s42255-021-00385-9)

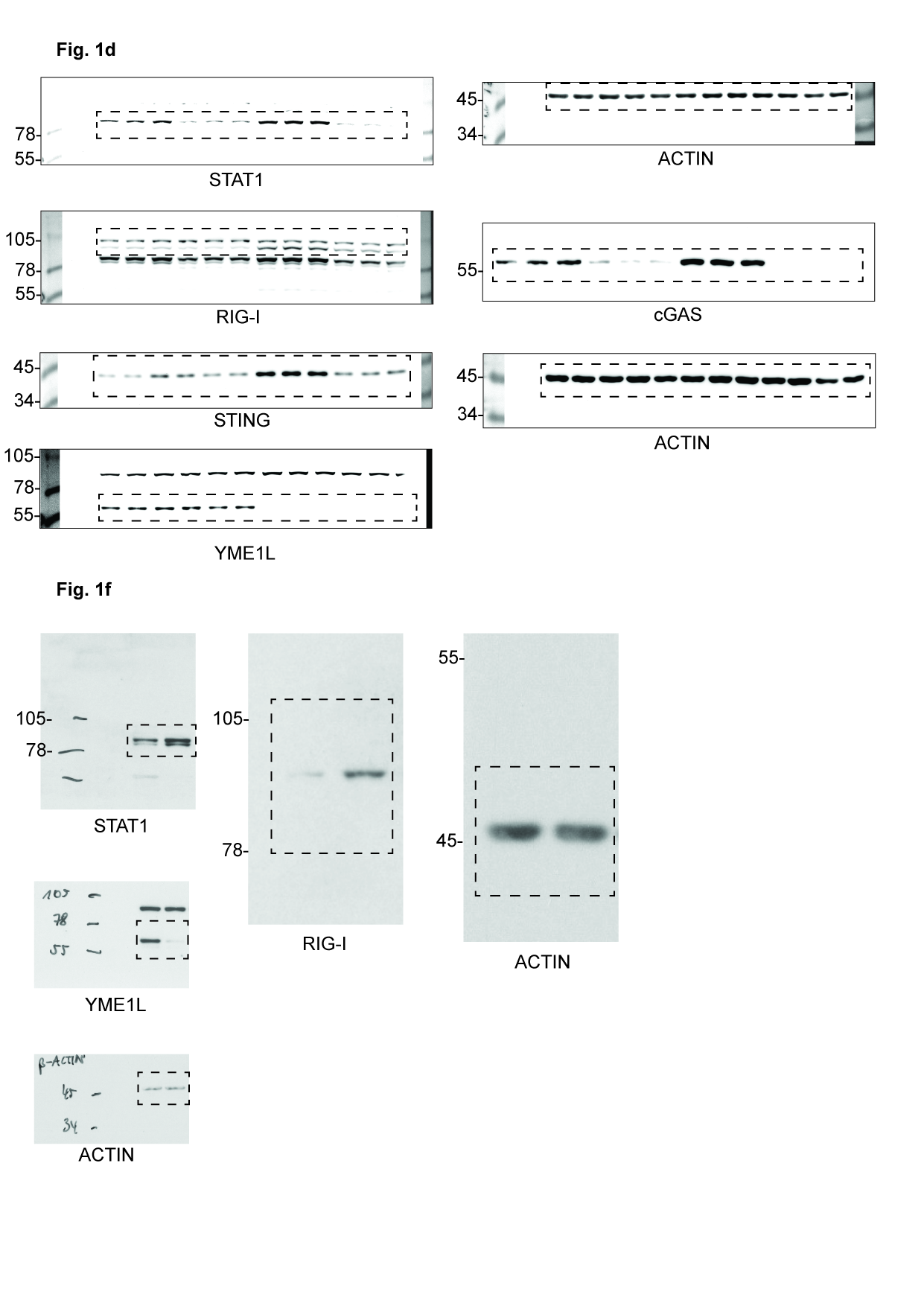

Supplement: Source Data Fig. 1 — Unprocessed immunoblots. [file 42255_2021_385_MOESM4_ESM.tif]

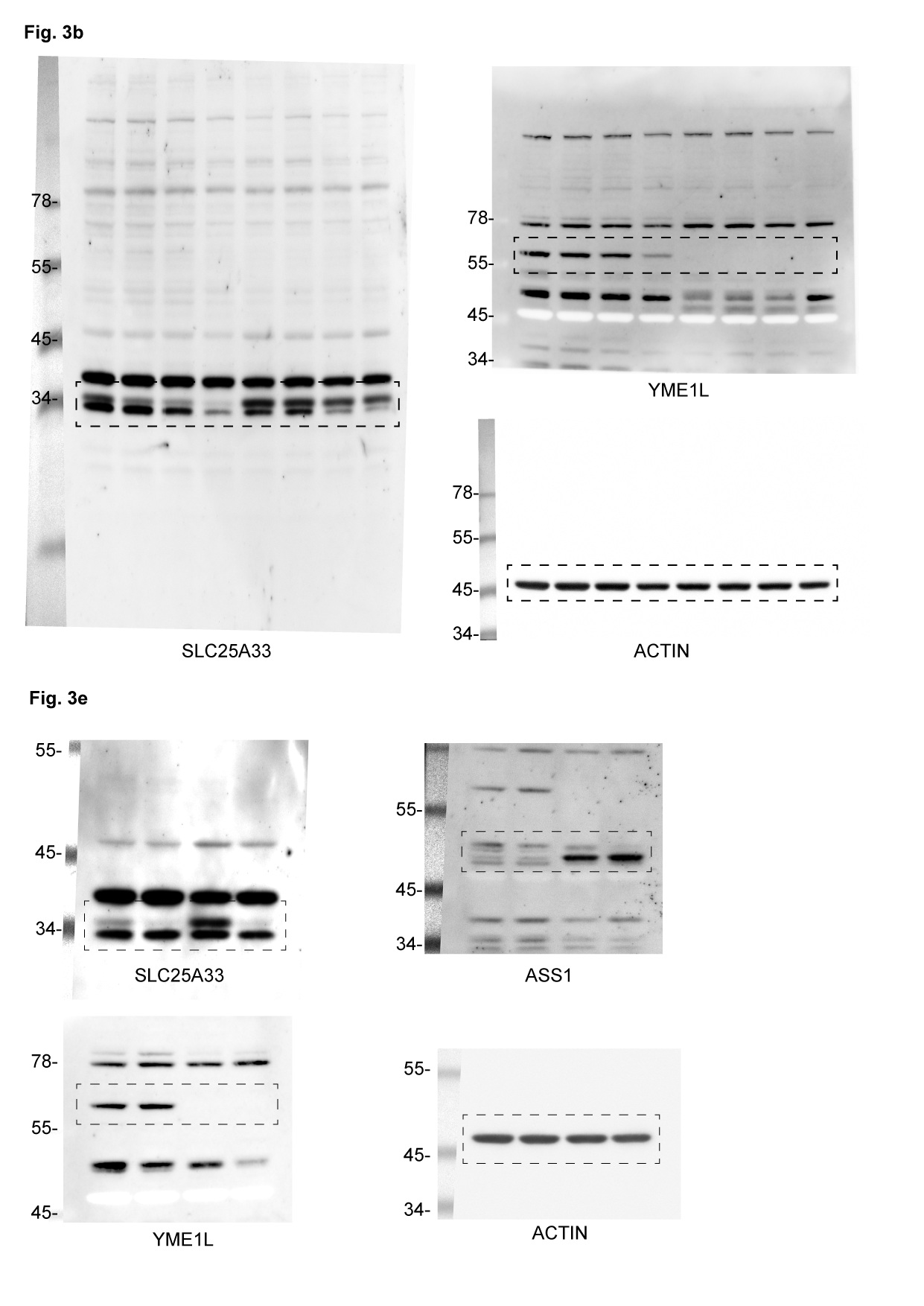

Supplement: Source Data Fig. 3 — Unprocessed immunoblots. [file 42255_2021_385_MOESM5_ESM.tif]

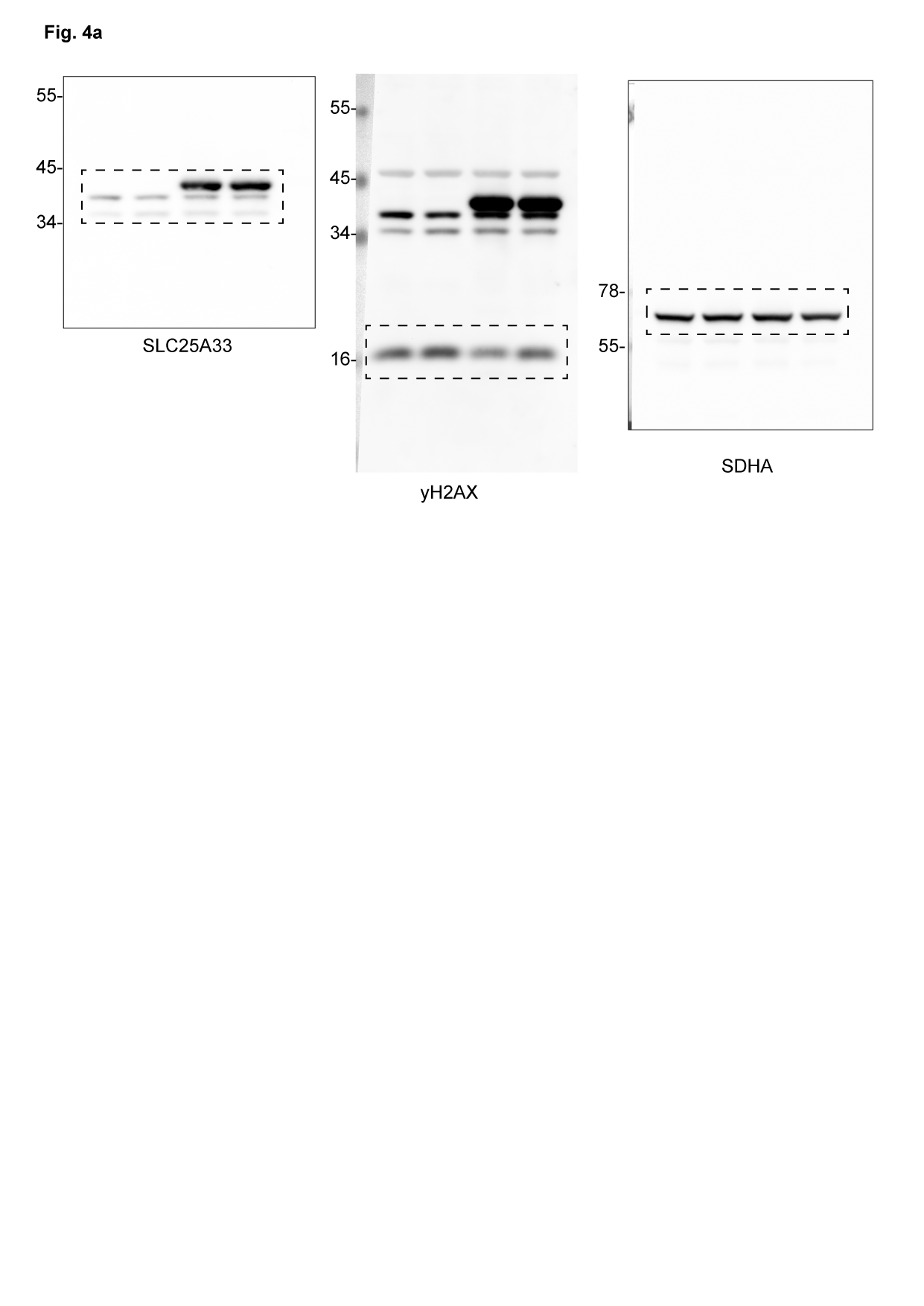

Supplement: Source Data Fig. 4 — Unprocessed immunoblots. [file 42255_2021_385_MOESM6_ESM.tif]

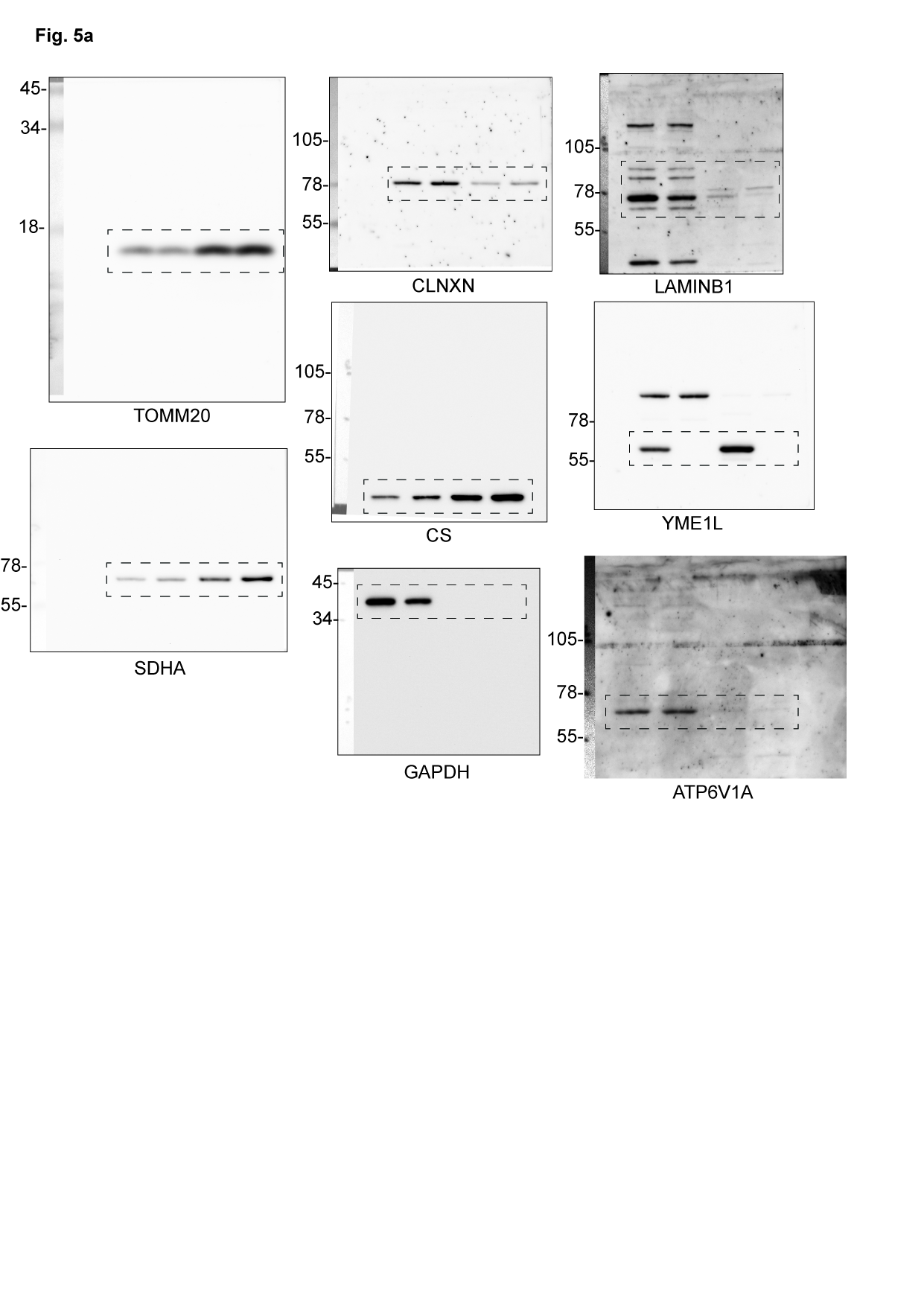

Supplement: Source Data Fig. 5 — Unprocessed immunoblots. [file 42255_2021_385_MOESM7_ESM.tif]

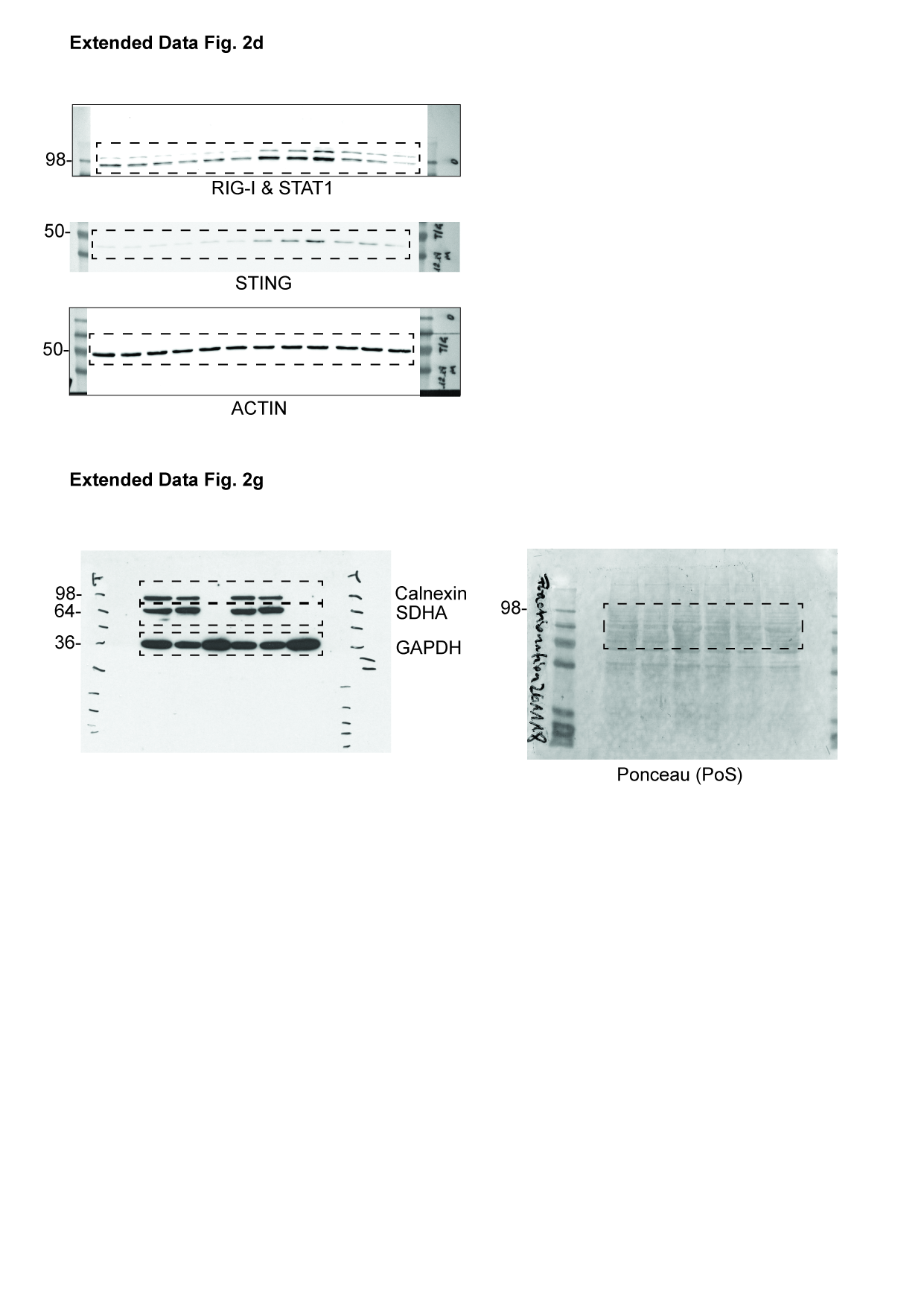

Supplement: Source Data Extended Data Fig. 2 — Unprocessed immunoblots. [file 42255_2021_385_MOESM8_ESM.tif]

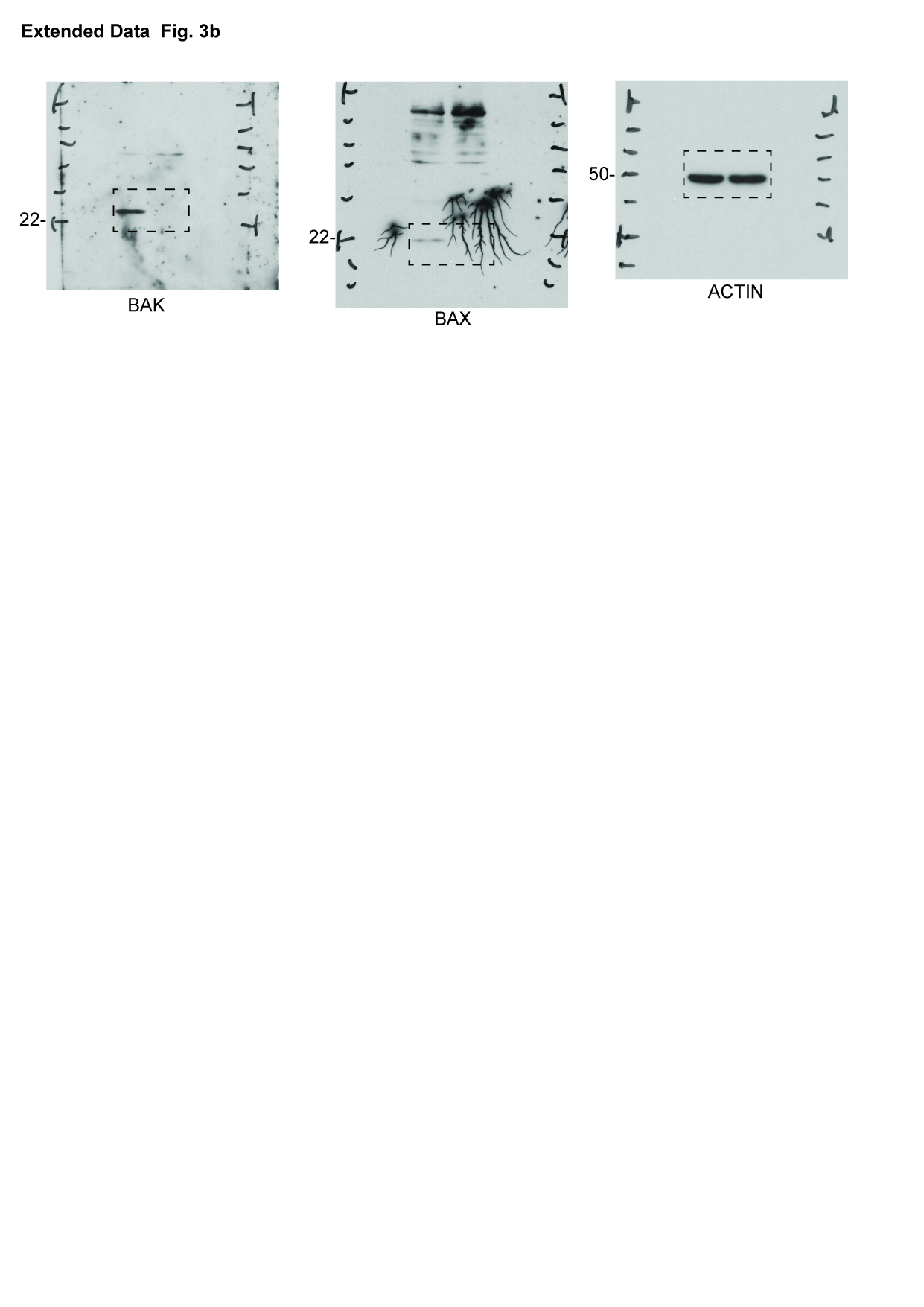

Supplement: Source Data Extended Data Fig. 3 — Unprocessed immunoblots. [file 42255_2021_385_MOESM9_ESM.tif]

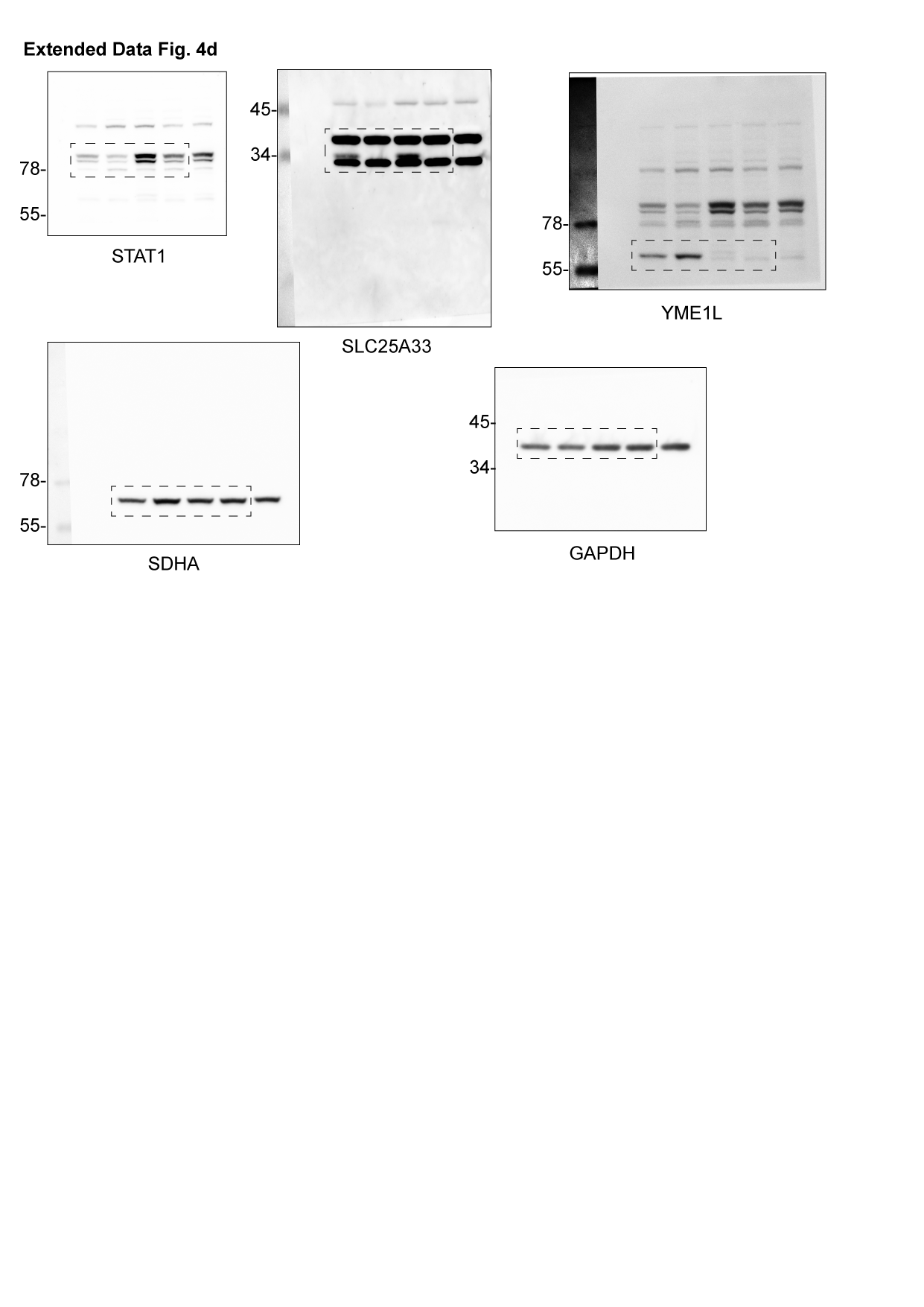

Supplement: Source Data Extended Data Fig. 4 — Unprocessed immunoblots. [file 42255_2021_385_MOESM10_ESM.tif]

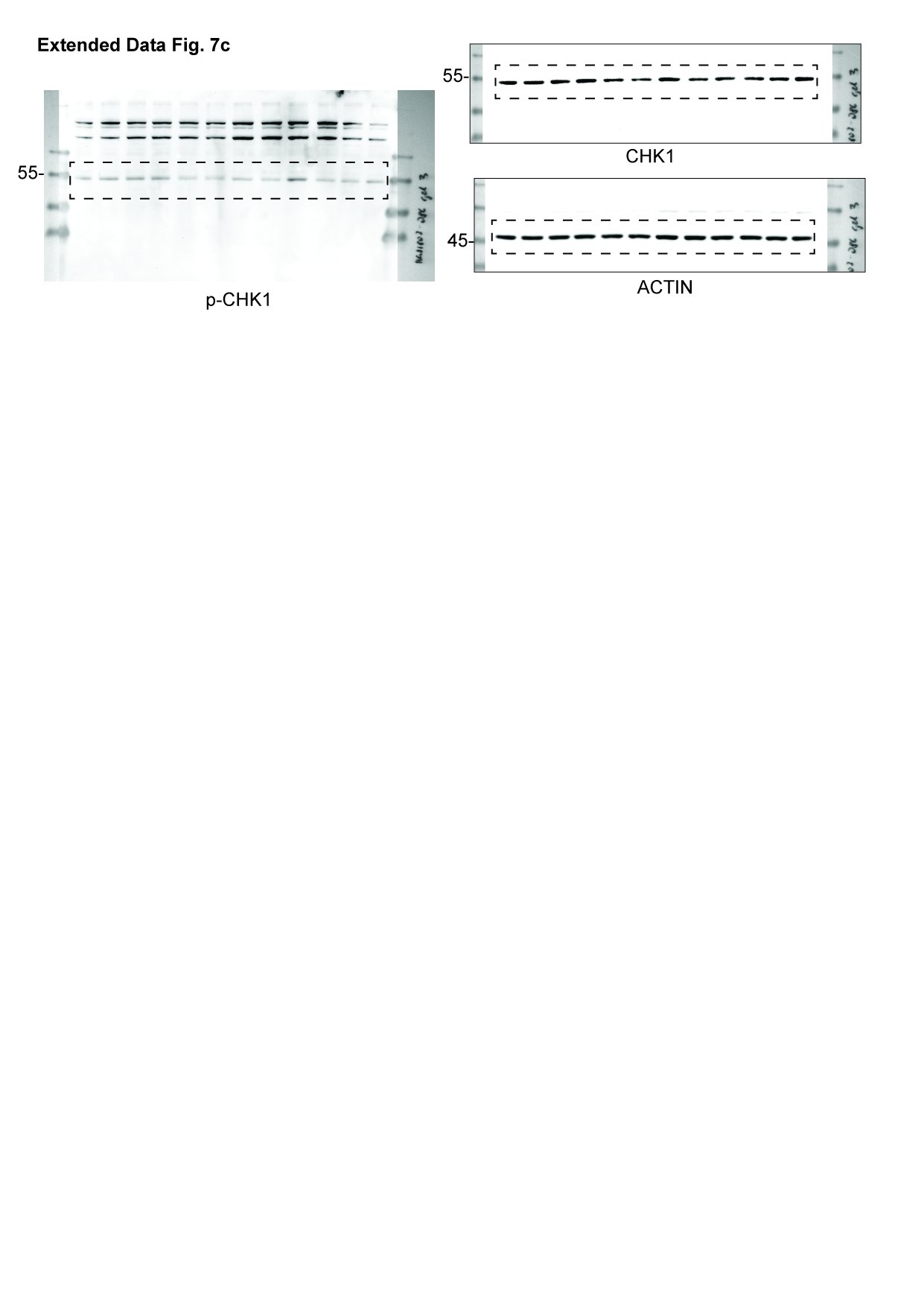

Supplement: Source Data Extended Data Fig. 7 — Unprocessed immunoblots. [file 42255_2021_385_MOESM11_ESM.tif]

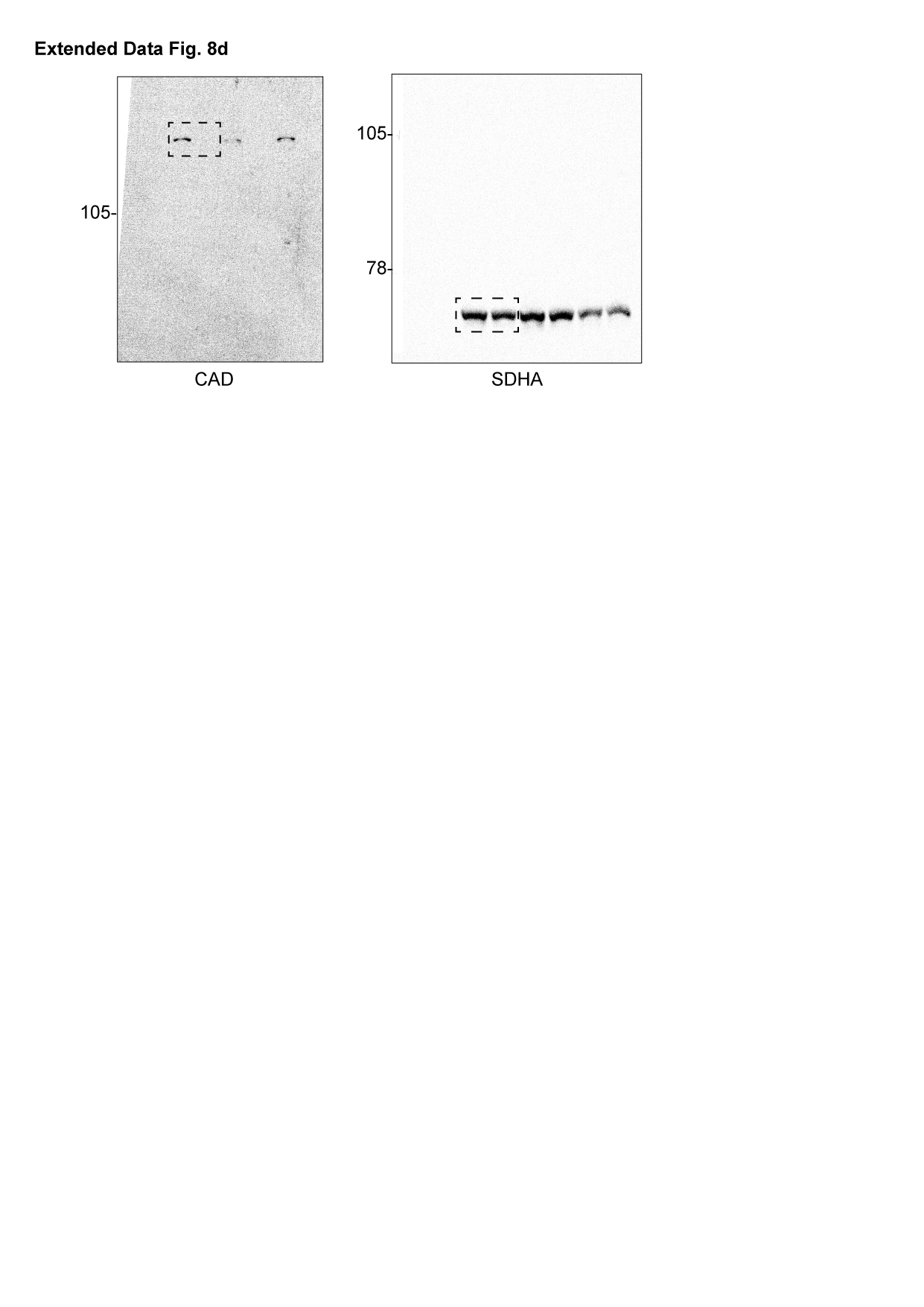

Supplement: Source Data Extended Data Fig. 8 — Unprocessed immunoblots. [file 42255_2021_385_MOESM12_ESM.tif]
